# Reprogramming signal transduction through a designer receptor tyrosine kinase

**DOI:** 10.1038/s42003-021-02287-8

**Published:** 2021-06-17

**Authors:** Tatphon Kongkrongtong, Yuka Sumigama, Teruyuki Nagamune, Masahiro Kawahara

**Affiliations:** 1 Department of Chemistry and Biotechnology, Graduate School of Engineering, The University of Tokyo, Tokyo, Japan; 2 Laboratory of Cell Vaccine, Center for Vaccine and Adjuvant Research (CVAR), National Institutes of Biomedical Innovation, Health and Nutrition (NIBIOHN), Osaka, Japan

**Keywords:** Synthetic biology, Protein design

## Abstract

Controlling signal transduction with artificial designer receptors is a promising approach to realize future medicine for intractable diseases. Although several functional artificial receptors have been reported by domain engineering, more sophisticated engineering within domains has yet to be thoroughly investigated. Here we demonstrate motif-based engineering of a receptor tyrosine kinase for reprogramming signal transduction. We design a scaffold-less tyrosine kinase domain that does not recruit any signal transducers but retains its kinase function. The resultant scaffold-less tyrosine kinase domain is linked to a tyrosine motif that recruits a target signaling molecule upon its phosphorylation. The engineered tyrosine motif–kinase fusion protein is further connected to a small molecule- or light-dependent dimerizing domain that can switch on the kinase activity in response to an external stimulus. The resultant designer receptors attain specific chemical- or photo-activation of signaling molecules of interest in mammalian cells. Thus, our designer receptor tyrosine kinase proves the possibility of rationally reprogramming intracellular signal transduction on a motif basis. The motif-based receptor engineering may realize tailor-made functional receptors useful in the fields of biology and medicine.

## Abstract (web summary)

Kongkrongtong et al. present motif-based engineering of tyrosine kinase receptor for reprogramming signal transduction. Here, by swapping internal tyrosine of receptor with those that bind intracellular STAT signaling proteins, they demonstrate that specificity of this receptor can be reprogrammed with this approach.

## Introduction

Signal transduction is initiated upon binding of ligands to receptors, followed by activation of many intracellular signaling molecules, part of which translocate to the nucleus to evoke cellular responses such as cell proliferation, differentiation, migration, and apoptosis^1–3^. The signaling events keep homeostasis of our body, and thus the abnormality of signaling causes pathogenesis^4,5^. Because of the attractiveness and importance, many investigators have endeavored to elucidate the mystery of intracellular signaling phenomena, and to date have identified most of the signaling molecules and pathways involved. The next-generation promising issue may be the artificial control of signaling events, and this paradigm shift will realize future medicine for intractable diseases.

Nowadays, there are several technologies which can rewire intracellular signaling pathways^6–10^. The intracellular and extracellular domains of receptors can be artificially replaced with functional domains to change the input and output as desired to produce receptors that do not exist in nature. One of the examples is a chimeric antigen receptor (CAR) consisting of a single-chain Fv (scFv) as the extracellular domain, a costimulatory signal domain and the signal transduction chain ζ of the CD3 complex downstream of T cell receptor as the intracellular domain^11^. Engineered T cells in which CAR is forcibly expressed (CAR-T cells) can recognize specific antigens on the surface of cancer cells, exert the function of T cells, and eradicate the tumor. Another example is a synthetic notch (SynNotch) receptor, in which the extracellular and intracellular domains of Notch is replaced with scFv and a transcription factor^12,13^. When expressed on the cell surface, SynNotch recognizes a specific antigen expressed on another cell surface in the extracellular domain and excises the transcription factor in the intracellular domain, leading to conditional gene expression by the excised transcription factor. Although such functional artificial receptors can be generated by relocation on a domain basis, more sophisticated engineering within the domain has yet to be thoroughly investigated^10^. In other words, if the artificial receptor can be engineered on a smaller unit basis such as a motif or amino acid basis, it will be possible to generate artificial receptors with diverse functions and enhanced signaling efficiency.

Our research team has focused on the activation mechanism of cytokine receptors and reprogramming of cellular signal transduction. Using the Janus kinase (JAK)-binding domain of a type-I cytokine receptor c-Mpl, and the tyrosine motif, which is a sequence around a dozen amino acid residues and recruits specific signaling molecules when phosphorylated, we successfully demonstrated specific activation of the target signaling molecules^14–16^. However, JAK, an endogenous tyrosine kinase, has a drawback that it constitutively activates STAT5 in a tyrosine motif-independent manner. In this study, we aim to design a new artificial receptor using a tyrosine kinase-included receptor that can be exogenously expressed instead of the JAK-based artificial receptors in order to avoid nonspecific STAT5 activation due to endogenous JAK. To attain this, we firstly design (i) a scaffold-less tyrosine kinase domain that does not recruit any signal transducers but retains its kinase function. Experimentally, we choose c-KIT, one of the receptor tyrosine kinases which are activated by dimerization, and modify the kinase domain of c-KIT by adding tyrosine-to-phenylalanine mutations on the signaling molecule-binding motifs. Next, the resultant scaffold-less tyrosine kinase domain is linked to (ii) a tyrosine motif that recruits a target signaling molecule upon its phosphorylation^17–20^. The engineered tyrosine motif-kinase fusion protein is further connected to (iii) a small molecule-dependent or light-dependent dimerizing domain that can switch on the kinase activity in an external stimulus-dependent manner. We verify whether the designer receptor tyrosine kinase (dRTK) created by the above-mentioned concept could activate the target signaling molecules, and whether the signaling intensity and pattern could be controlled by modulating the intensity and time of the external stimuli. Consequently, our designer receptor proved the capability of sophisticated receptor engineering on a motif or amino acid basis. dRTK could be a useful tool for elucidating signaling pathways and provide a venue for the rational design of artificial receptors useful for many applications in cell therapy and synthetic biology.

## Results

### Construction of a designer receptor tyrosine kinase

**Fig. 1:**
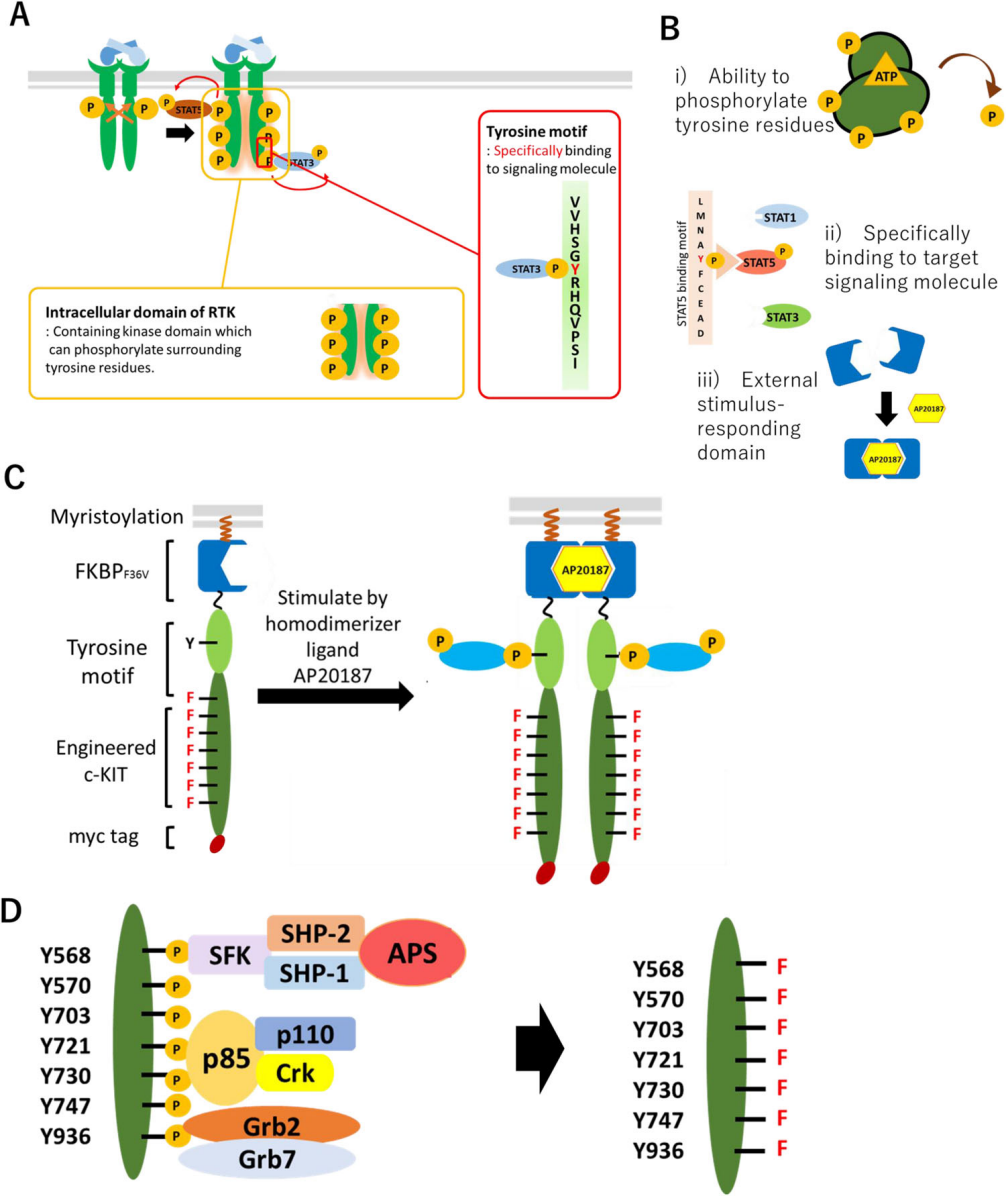
Schematic illustration of designer receptors. **A** Ligand binding to the receptor tyrosine kinase (RTK) leads to activation of the intracellular kinase domain. The intracellular domain of c-KIT is engineered in this study. A tyrosine motif is a short sequence which is derived from another receptor. **B** The designer receptors in this study consist of 3 parts; (i) an engineered tyrosine kinase which has an ability to phosphorylate the tyrosine motif, (ii) a tyrosine motif, which specifically binds to a target signaling molecule when phosphorylated, and (iii) an external stimulus-responding dimerizing domain (e.g., AP20187-responsive FKBP_F36V_). **C** Illustration of the activation process of the designer receptor. FKBP_F36V_, a tyrosine motif, and the engineered c-KIT are linked together to create a designer receptor. The designer receptor dimerizes when stimulated by AP20187. **D** The kinase domain of wild-type c-KIT functions as a scaffold domain which recruits many intracellular signaling molecules. To obtain the domain which functions only as a kinase, the 7 tyrosine residues which are known as a scaffold for recruiting signaling molecules are mutated to phenylalanine.

By considering the molecular architecture and activation mechanism of receptor tyrosine kinases (Fig. 1A), a designer receptor that activates only the signal transducer of interest was created using the following components; (i) the kinase domain to phosphorylate a tyrosine residue within the tyrosine motif, (ii) a tyrosine motif that binds specifically to signal transducers upon its phosphorylation, and (iii) FKBP_F36V_, which dimerizes in a ligand-dependent manner to control the on/off of receptor activation^21^ (Fig. 1B).

The designer receptor, made from the above-described design strategy, dimerizes upon ligand stimulation. Once the kinase domain is activated, it phosphorylates the tyrosine residue of the tyrosine motif, and then the target signal transducer is recruited via the phosphotyrosine binding (PTB) domain or Src homology 2 (SH2) domain^22^. The recruited signal transducer is phosphorylated and activated by the kinase domain, which leads to downstream signal transduction cascades.

Fig. 1C shows the architecture of our firstly constructed designer receptor. To mimic the wild-type receptor, a myristoylation signal sequence was appended at the N-terminus of the designer receptor for localization on the inner leaflet of the plasma membrane. The c-KIT intracellular domain was used as a kinase domain. Seven tyrosine residues, which were known to bind to signal transducers^23,24^ on the intracellular domain, were mutated to phenylalanine residues (a 7YF mutant), in order to create a domain which could not recruit signal transducers but retain an ability to phosphorylate tyrosine residues (Fig. 1D). The amino acid sequences of all designer receptors constructed in this study are summarized (Supplementary Figs. 1–6). The positions of the seven tyrosine residues in the c-KIT intracellular domain are shown (Supplementary Fig. 7).

### Juxtamembrane engineering yields a functional designer receptor

To investigate whether the designer receptor is functional, interleukin-3-dependent murine pro-B Ba/F3 cells were employed for expression. Ba/F3 cells are a useful host for detecting designer receptor-derived signaling, because the cellular signaling activity can be significantly diminished in the absence of IL-3. Furthermore, Ba/F3 cells are easy to handle because of suspension cells, and the retroviral gene transduction efficiency is high, making it easy to obtain stable transductants. Ba/F3 cells were transduced with a retroviral vector encoding the designer receptor and puromycin-resistance genes, followed by puromycin selection to obtain stable transductants. The cells were stimulated with an FKBP_F36V_-homodimerizing ligand AP20187, and the phosphorylation of target signal transducer (STAT3 in this experiment) was confirmed by western blotting. However, STAT3 phosphorylation was not observed (Supplementary Fig. 8), indicating that the kinase function was not maintained in the engineered 7YF mutant.

**Fig. 2:**
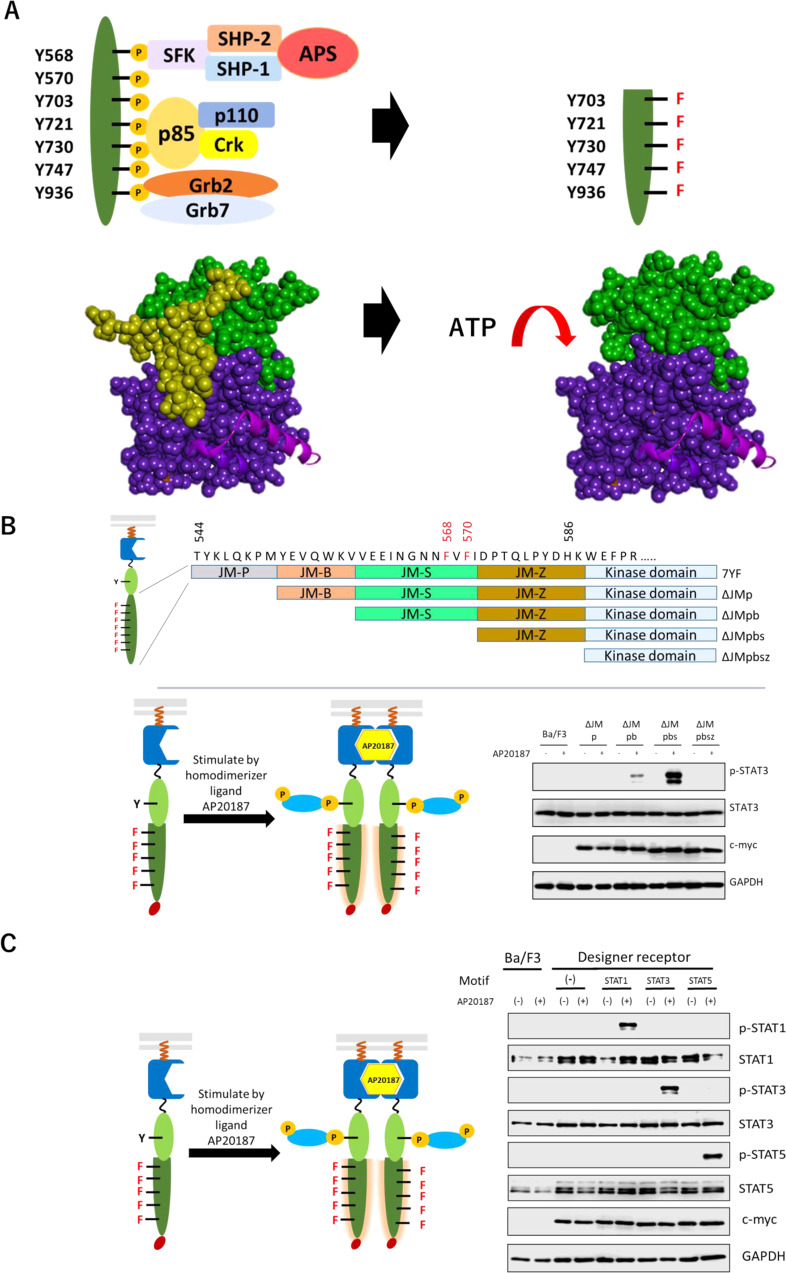
Optimizing juxtamembrane domain of c-KIT. **A** Deletion of the juxtamembrane domain of the intracellular domain of c-KIT makes ATP easier access to the activation center. The color of space-filling figure denotes: yellow, juxtamembrane domain; green, proximal kinase domain; purple, distal kinase domain. **B** The juxtamembrane domain consists of JM-p, JM-b, JM-s, and JM-z. By gradually deleting each domain, four deletion mutants ΔJMp, ΔJMpb, ΔJMpbs, and ΔJMpbsz were created. The STAT3-binding motif was used as a tyrosine motif located at the N-terminus of the engineered c-KIT. In western blotting, parental Ba/F3 and the transduced cells were unstimulated (−) or stimulated with 50 nM AP20187(+). Phospho-STAT3, whole STAT3, c-myc tag (chimeric receptor expression), and GAPDH were detected using corresponding primary antibodies. **C** The three tyrosine motifs which specifically bind to STAT1, STAT3, and STAT5, respectively, were used as a tyrosine motif located at the N-terminus of the engineered c-KIT. Phospho-STAT1/3/5 and whole STAT1/3/5 were detected using corresponding primary antibodies. The full uncropped blot images are provided as Supplementary Fig. 17.

In order to restore the kinase activity of the engineered c-KIT, genetic modification of the juxtamembrane domain (JMD), which involves in the regulation of kinase activity^23^, was considered (Fig. 2A). In the inactivated state of c-KIT, V-shaped JMD prevents the kinase activation loop from extension^25^. By phosphorylating Y568 and Y570 on JMD, JMD straightens, and then the activation loop extends, which allows ATP access to the active center and leads to the active state^23^.

Because Y568 and Y570 on JMD were mutated to phenylalanine residues in the engineered 7YF mutant, we hypothesized that structural changes triggered by phosphorylation would not occur, which hampered switching to the activated state. In order to solve this problem, variants in which the segments of JMD were gradually deleted were constructed.

Because JMD has four segments^23^, JM-P, JM-B, JM-S, and JM-Z, the segment-deletion mutants (ΔJMp, ΔJMpb, ΔJMpbs, and ΔJMpbsz) were constructed by gradually deleting each segment (Fig. 2B). Consequently, phosphorylation of the target signal transducer STAT3 was clearly observed in the ΔJMpbs mutant, indicating that the kinase activity was restored by deleting the N-terminal three segments of JMD. A time-dependent stimulation experiment revealed that STAT3 was phosphorylated by ligand stimulation from 1 to 60 min (Supplementary Fig. 9). STAT3 was drastically activated in 3 min, and the activation level decreased after 60 min, indicating signal attenuation. The STAT3 activation in the ΔJMpbs mutant was dependent solely on the STAT3-binding motif, since the Y to F mutation of the STAT3-binding motif completely abolished the STAT3 phosphorylation (Supplementary Fig. 10). Furthermore, the STAT3 activation was inhibited by addition of a tyrosine kinase inhibitor Dasatinib, indicating that the STAT3 was activated through the activation of the engineered c-KIT mutant ΔJMpbs (Supplementary Fig. 11). Surprisingly, the myristoylation signal sequence was not required for functionality of the ΔJMpbs mutant (Supplementary Fig. 12). In addition, when the STAT3-binding tyrosine motif was replaced with either STAT1- or STAT5-binding motif, specific activation of these STATs was also attained (Fig. 2C). Therefore, the ΔJMpbs mutant was used for the following experiments.

### Scanning activatable tyrosine motif positions within engineered c-KIT

Next, we investigated whether there might be other positions capable of inserting a tyrosine motif on the engineered kinase. Wild type c-KIT has many signal transducers-binding sites on the kinase insert domain (KID). Furthermore, since KID is a flexible region^23^, we hypothesize that insertion of an extrinsic tyrosine motif at KID does not affect the kinase activity of the engineered kinase. The C-terminal tail region is also flexible and easily accessible by signal transducers.

**Fig. 3:**
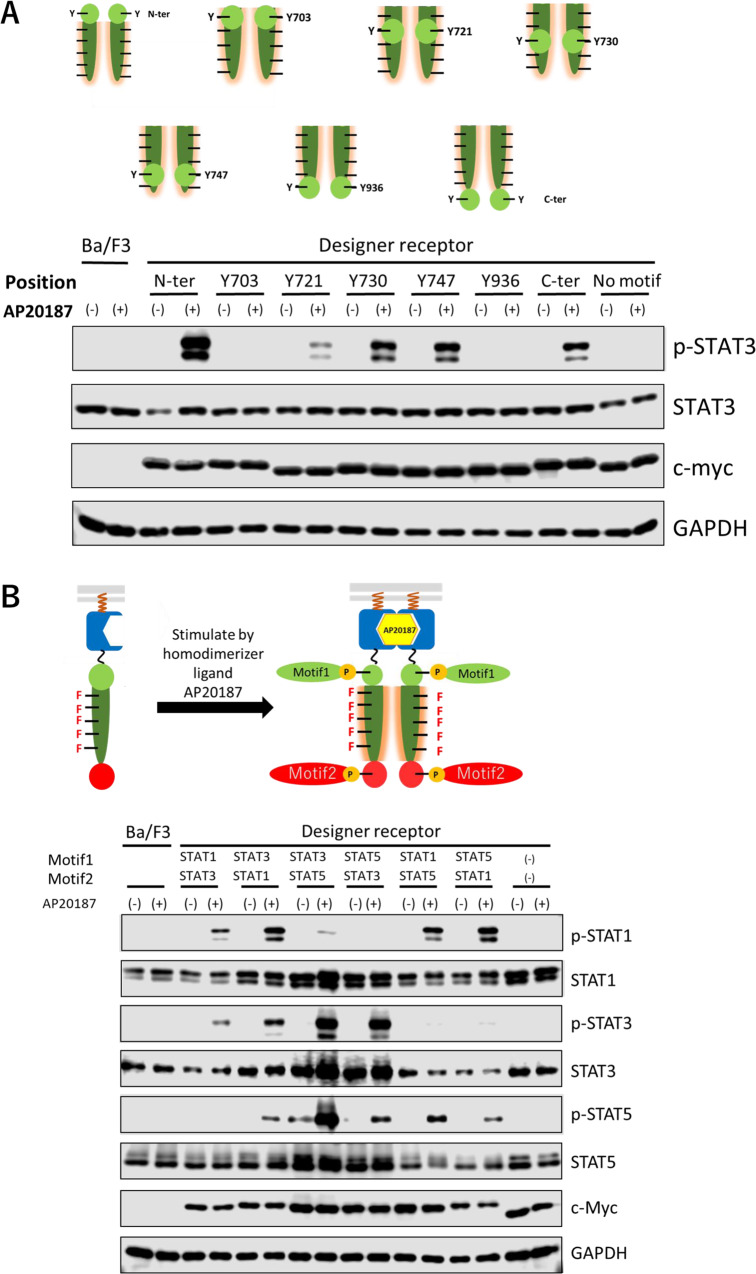
Simultaneous activation of multiple signaling molecules using multiple tyrosine motifs. **A** Exploring appropriate positions of tyrosine motifs located in the engineered c-KIT. The STAT3-binding motif was located at the N-terminus (N-ter), Y703, Y721, Y730, Y747, or Y936 or C-terminus (C-ter) of the engineered c-KIT. In western blotting, parental Ba/F3 and the transduced cells were unstimulated (−) or stimulated with 50 nM AP20187(+). Phospho-STAT3, whole STAT3, c-myc tag (chimeric receptor expression), and GAPDH were detected using corresponding primary antibodies. **B** Two tyrosine motifs, motif1 and motif2, were added to the N-terminus and C-terminus of the engineered c-KIT, respectively. Phospho-STAT1/3/5, whole STAT1/3/5, c-myc tag (chimeric receptor expression), and GAPDH were detected using corresponding primary antibodies. The full uncropped blot images are provided as Supplementary Fig. 17.

Thus, Y703, Y721, Y730, Y747 at KID and Y936 at the C-terminus of the kinase domain together with their surrounding amino acid residues were replaced with the STAT3-binding tyrosine motif so that the number of amino acid residues remain the same. We also constructed a C-terminal appended variant (C-ter) and compared the phosphorylation level of the target signal transducer STAT3 with the original N-terminal one (N-ter) (Fig. 3A). As a result, Y730, Y747, and C-ter variants exhibited sufficient but weaker phosphorylation levels of STAT3 than the original N-ter. The Y721 variant induced much lower phosphorylation level of STAT3, whereas no phosphorylation was observed in the Y703 and Y936 variants. The N-ter, Y730, Y747, and C-ter variants were able to phosphorylate STAT1 and STAT5 when the STAT3-binding motif was replaced with STAT1- and STAT5-binding motif (Supplementary Figs. 13 and 14).

### Reconstituting a signaling scaffold with two tyrosine motifs

Next, we aimed to develop designer receptors that contain two tyrosine motifs and activate two target signal transducers simultaneously. The tyrosine motifs were inserted at the N- and C-termini of the engineered kinase where phosphorylation was clearly confirmed. Using STAT1-, STAT3-, and STAT5-binding tyrosine motifs, a total of six designer receptors were created using these combinations (Fig. 3B).

As a result, phosphorylation of two target signal transducers was confirmed. In the chimeric receptor sample “STAT1-STAT3”, in which the STAT1- and STAT3-binding motifs are inserted in the N- and C-termini of the engineered kinase in this order, specific phosphorylation of the targets STAT1 and STAT3 was attained. Samples “STAT5-STAT3”, “STAT1-STAT5”, and “STAT5-STAT1” were also able to clearly phosphorylate the target signal transducers. In sample “STAT3-STAT1”, phosphorylation of the targets STAT3 and STAT1 was attained, but nonspecific phosphorylation of STAT5 was also observed at the same time. Although further verification is required, the phenomenon that STAT5 is also activated simultaneously when STAT1 is strongly activated is often observed in this study. In sample “STAT3-STAT5”, phosphorylation of STAT3 and STAT5 was attained, but the activation intensity of STAT5 was too strong, so that the phosphorylation of non-target STAT1 was also observed.

From this result, the designer receptors were able to activate the target signal transducers as desired. In order to insert the second tyrosine motif, a position that does not affect the kinase activity and capable to bind signal transducers is desirable. In the case of engineered c-KIT, it was possible to activate two signal transducers simultaneously by inserting tyrosine motifs at the N- and C-termini of the kinase domain. In addition, tyrosine motifs can be inserted into Y730 and Y747 in the KID region, and there is a possibility that a third tyrosine motif can be inserted.

To examine whether the designer receptors could make effects on cellular phenotype, the proliferation-inducing activity was measured for Ba/F3 transductants expressing the designer receptors that activate either STAT1, STAT3, or STAT5, and those that activate two of them (Supplementary Fig. 15). As a result, the receptor with the STAT3- or STAT5-binding motif induced cell proliferation, while the receptor with the STAT1-binding motif did not. For the two-motif receptors, the proliferation was improved in the combination of the STAT3- and STAT5-binding motifs, while the proliferation levels were lowered in the combination with the STAT1-binding motif. These results demonstrate that the designer receptors can be applied to cell fate control.

### Photo-activation of a target signaling molecule

**Fig. 4:**
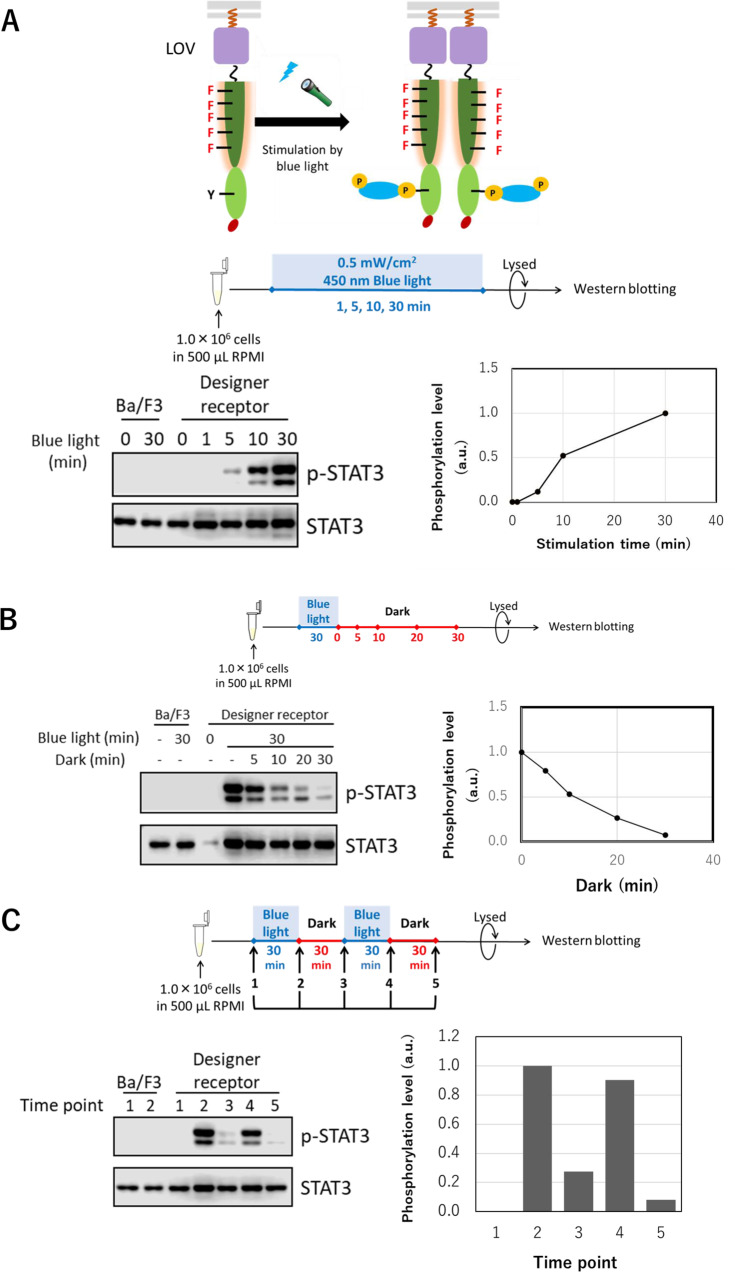
Light-inducible designer receptor. Parental Ba/F3 and the transduced cells were unstimulated (0) or stimulated with blue light (0.5 mW/cm^2^, 450 nm) or kept with no light (dark). **A** The LOV domain, which dimerizes upon irradiation of blue light, was substituted for FKBP_F36V_ in the chimeric receptor with the STAT3-binding motif at the C-terminus to obtain a blue light-sensitive designer receptor. Western blotting was conducted for detecting phosphorylated and whole STAT3. The phosphorylation level of STAT3 was digitized by normalizing the whole STAT3 level of each sample, and the graph shows the relationship between the STAT3 phosphorylation level and stimulation time. **B** The STAT3 phosphorylation level decreased depending on incubation time in the dark. **C** The STAT3 phosphorylation level was reversibly controlled by switching light on/off. The full uncropped blot images are provided as Supplementary Fig. 17.

In the chemical-inducible system using FKBP_F36V_, it was difficult to control the signal activation intensity and pattern. Therefore, we created a new designer receptor that can be controlled spatiotemporally by using a photosensitive *V. frigida* Aureochrome LOV domain^26,27^ instead of FKBP_F36V_ (Fig. 4). Similar to the FKBP_F36V_-based designer receptors, a myristoylation signal sequence was added for localization on the plasma membrane, and the LOV domain was used instead of FKBP_F36V_. When the STAT3-binding motif was located at C-ter, Y730, and Y747, STAT3 was successfully activated by light irradiation (Supplementary Fig. 16). Therefore, C-ter position was chosen for the following experiments.

First, the light irradiation time dependency of phosphorylation levels of STAT3 was examined (Fig. 4A). The irradiation time was 1, 5, 10, and 30 min, respectively. The dependence of phosphorylation levels on the stimulation time was observed.

Next, it was examined whether the light-inducible designer receptor could be switched off when incubated in a dark state (Fig. 4B). After 30 min of light irradiation, the phosphorylation level gradually decreased depending on the incubation time in the dark state. The phosphorylation level was halved after 10 min of stimulation, and the phosphorylation almost disappeared after 30 min.

Finally, it was examined whether the phosphorylation level increased again when stimulated again after phosphorylation was attenuated, and decreased again after the stimulation (Fig. 4C). It was confirmed that phosphorylation was reversibly turned on and off depending on light irradiation. When comparing before stimulation (1) and after stimulation (2), the phosphorylated band appeared remarkably. Thereafter, the decay of the phosphorylation level was confirmed by incubation for 30 min (3) in a dark state. When stimulated again (4), the phosphorylation level increased to about 90% of the first stimulation. Furthermore, the same attenuation was confirmed even when incubated for 30 min (5) in a dark state. It was proved that the designer receptor can control the activation pattern of the target signal transducer in high spatiotemporal resolution by light irradiation.

## Discussion

In this study, we designed an artificial receptor that can specifically activate target signal transducers. For this purpose, we combined a tyrosine motif that specifically recruits the target signal transducer upon phosphorylation, and a kinase domain which phosphorylates tyrosine residues on the receptor chain. The intracellular domain of c-KIT was engineered to obtain a modified kinase domain that only functions as a kinase without a scaffolding function. Due to the proximity of the modified kinase domain and the tyrosine motif, the kinase domain phosphorylated the tyrosine motif, then recruited and activated the target signal transducer. Our results suggest that intracellular signal transduction pathways can be rationally designed by our designer receptor. In addition, signal transducers of interest were activated by placing corresponding tyrosine motifs on the designer receptor. Furthermore, we identified multiple positions for inserting tyrosine motifs on the designer receptor, where multiple signal transducers can be activated simultaneously. These results demonstrated that our newly constructed designer receptor tyrosine kinase can reconstitute signal transduction properties.

We succeeded in photoactivation by replacing the small molecule-inducible dimerizing domain with the light-sensitive domain in the designer receptor. Therefore, the artificial receptor can be used not only to control a large number of cells simultaneously but also to spatiotemporally control signals at single-cell levels. Grusch et al. performed an excellent research using wild-type FGFR and identified the LOV domain that can be used for receptor engineering^28^. In addition, the authors performed tyrosine-to-phenylalanine substitution of FGFR to identify which motifs are important for the Ras/MAPK pathway. On the other hand, using their LOV, we succeeded in imparting new signaling specificity to an engineered c-KIT by adding tyrosine motifs of interest. This is a novel aspect of our research compared to their research. To the best of our knowledge, this is the first report that RTK has been artificially designed in the unit of tyrosine motifs, which are much smaller than domains. Thus, this study provides useful information for the artificial design of functional signaling receptors.

In order to design functional intracellular proteins, it is necessary to know the structure of the protein as well as the function of each domain. It has been reported that the intracellular domain of c-KIT has seven tyrosine residues to which signal transducers are recruited^23^. To eliminate the scaffold function, the tyrosine residues need to be replaced with phenylalanine residues so that they cannot be phosphorylated. However, this mutational approach led to the loss of the tyrosine kinase activity. Therefore, we modified the juxtamembrane domain, which critically involves in the activation of c-KIT, to produce a domain capable of maintaining the tyrosine kinase activity while substituting phenylalanine residues. Phosphorylation of Y568 and Y570 in the juxtamembrane domain is important for the activation of c-KIT^23^. The crystallographic structural data suggest that phenylalanine substitution of these two tyrosine residues might fail to induce the structural change of the juxtamembrane domain, indicating that the c-KIT could not transition to the active state. In the c-KIT activation mechanism, the juxtamembrane domain is extended by phosphorylation, and then ATP becomes possible to access to the active center of c-KIT, thereby being activated. Therefore, the juxtamembrane domain (aa 544–571) that acts like a lid was deleted. Then, even if Y568 and Y570 were not phosphorylated, a domain capable of maintaining the kinase activity by dimerization was obtained.

Substitution of a tyrosine motif for those which c-KIT originally recruits signal transducers did not always result in successful activation of a target signal transducer. Because the tyrosine motif should be displayed on the protein surface, the appropriate positions to be inserted should be predicted and identified from the crystallographic structural data. Although we focused on STAT-binding motifs in this study, there are tyrosine motifs that preferentially bind to specific signaling molecules other than STATs. Therefore, in order to activate a target signal transducer other than STATs, the corresponding tyrosine motif may be selected. However, as revealed in this study, the specificity of the tyrosine motif should be carefully evaluated because the motif is placed in a different environment depending on the insertion site within the c-KIT. This notion is also true for light-inducible designer receptors, in which a tyrosine motif attached just downstream of the LOV domain was unfunctional. The C-terminal region of LOV may hide the following peptide sequences and would not be suitable as a motif insertion site.

In summary, our designer receptor proves the possibility of rationally designing intracellular functional proteins on a motif basis. The motif-based receptor engineering will realize tailor-made functional receptors in the future and open new possibilities in the fields of biology and medicine.

## Materials and methods

### Plasmid construction

The amino acid sequences of all designer receptors constructed in this study are summarized in Supporting Information (Supplementary Figs. 1–6). All of the designer receptors were encoded by replacing the stuffer sequence of a retroviral expression plasmid pMK-stuffer-IPTG^29^, which contains an internal ribosomal entry site (IRES)–puromycin resistance gene cassette. In brief, pMK-stuffer-IPTG was linearized by PCR and fused to the PCR-amplified insert sequence utilizing an In Fusion HD enzyme (Takara Bio, Shiga, Japan). For mutagenesis, PCR was performed using PrimeSTAR Mutagenesis Basal Kit (Takara Bio). The plasmid products were subcloned by transformation of an *E. coli* strain NEB Turbo (New England Biolabs, Ipswich, MA) and picking up isolated colonies. The culture of each colony was expanded for extracting the plasmid and sequencing.

### Cell culture

The condition of cell culture was described in a previous study^29^. In brief, Ba/F3 cells were cultured at 37 °C under 5% CO_2_ in RPMI 1640 medium (Nissui Pharmaceutical, Tokyo, Japan) supplemented with 10% fetal bovine serum, 1 ng/ml murine IL-3 (R&D Systems, Cambridge, MA), 100 U/ml penicillin and 100 µg/ml of streptomycin (Thermo Fisher Scientific, Waltham, MA). Retroviral packaging Plat-E cells were cultured at 37 °C under 10% CO_2_ in Dulbecco’s modified Eagle’s medium (DMEM) (Nissui Pharmaceutical) supplemented with 10% fetal bovine serum, 1 µg/ml puromycin (Sigma-Aldrich, St. Louis, MO), and 10 µg/ml blasticidin (Kaken Pharmaceutical, Tokyo, Japan).

### Transduction and selection of Ba/F3 cells

The procedure of retroviral transduction of Ba/F3 cells was described in a previous study^29^. In brief, Plat-E cells were transfected with the constructed plasmids using Lipofectamine LTX (Thermo Fisher Scientific) according to the manufacturer’s protocol. After 48 h, Ba/F3 cells were transduced with the viral supernatant by using RetroNectin (Takara Bio, Shiga, Japan) according to the manufacturer’s protocol. Two days after transduction, the cells were selected by 2 µg/ml puromycin.

### Stimulation of cells

The designer receptor-expressing cells were washed three times by PBS, then cultured in the medium without IL-3 for 5 h before stimulation. The cells (1.0 × 10^6^ cells) were collected in a microtube by centrifugation at 380 *g*. For chemical stimulation, 500 μl of RPMI medium supplemented with 50 nM AP20187 (Takara Bio) was mixed with the cells pellet, then keep at 37°C for 15 min. For light stimulation, cells were exposed with 450 nm blue light at 0.5 mW/cm^2^ at 37 °C for appropriate time periods.

### Signaling analysis

The protocol for signaling analysis was described previously^14^. In brief, the stimulated cells (10^6^ cells) were washed immediately by ice-cold PBS containing 2 mM Na_3_VO_4_ twice, lysed with 100 μl of lysis buffer (20 mM HEPES (pH 7.5), 150 mM NaCl, 10% glycerol, 1% Triton X-100, 1.5 mM MgCl_2_, 1 mM EGTA, 10 μg/ml aprotinin, 10 μg/ml leupeptin), and incubated on ice for 10 min. After centrifugation at 21,500 *g* for 10 min at 4°C, the supernatant was mixed with 33 μl of 4 x Laemmli’s buffer and boiled. The cell lysates were loaded to SDS-PAGE to analyze the signaling properties by western blotting. Following is the list of the rabbit primary antibodies used in this study: anti phospho-STAT1 (Y701)(Cell Signaling Technology, Danvers, MA), anti STAT1 (Cell Signaling Technology, Danvers, MA), anti phospho-STAT3 (Y705) (Cell Signaling Technology), anti STAT3 (Santa Cruz Biotechnology, Santa Cruz, CA), anti phosph-STAT5 (Y694) (Cell Signaling Technology), anti STAT5 (Santa Cruz Biotechnology), anti GAPDH (Santa Cruz Biotechnology), anti c-myc tag (BETHYL, Montgomery, TX). Horseradish peroxidase-conjugated anti rabbit IgG (Thermo Fisher Scientific) was used as a secondary antibody.

### Reporting summary

Further information on research design is available in the Nature Research Reporting Summary linked to this article.

## Supplementary information

Supplementary Information

Description of Supplementary Files

Supplementary Data 1

Reporting Summary

## Data Availability

All data generated or analysed during this study are included in this published article and its supplementary information. The raw data for Fig. 4 are provided as Supplementary Data 1.
