# Supplementary material for: Reprogramming signal transduction through a designer receptor tyrosine kinase
Source: Commun Biol. 2021 Jun 17;4:752. doi: 10.1038/s42003-021-02287-8 (PMC8211861; doi:10.1038/s42003-021-02287-8)

# Supplementary Information

## Reprogramming signal transduction through a designer receptor tyrosine kinase

Tatphon Kongkrongtong[1], Yuka Sumigama[1], Teruyuki Nagamune[1], Masahiro Kawahara[1,2,*]

## Table of Contents

1. **Supplementary Figure 1** ........................................ S2
2. **Supplementary Figure 2** ........................................ S3-S4
3. **Supplementary Figure 3** ........................................ S5
4. **Supplementary Figure 4** ........................................ S6-S8
5. **Supplementary Figure 5** ........................................ S9
6. **Supplementary Figure 6** ........................................ S10
7. **Supplementary Figure 7** ........................................ S11
8. **Supplementary Figure 8** ........................................ S12
9. **Supplementary Figure 9** ........................................ S13
10. **Supplementary Figure 10** ........................................ S14
11. **Supplementary Figure 11** ........................................ S15
12. **Supplementary Figure 12** ........................................ S16
13. **Supplementary Figure 13** ........................................ S17
14. **Supplementary Figure 14** ........................................ S18
15. **Supplementary Figure 15** ........................................ S19
16. **Supplementary Figure 16** ........................................ S20
17. **Supplementary Figure 17** ........................................ S21-S33

STAT1 binding motif

PTSFGYDKPHVL

Derivation of motif: Interferon $\gamma$ receptor 1

STAT3 binding motif

VVHSGYRHQVPS

Derivation of motif: gp130

STAT5 binding motif

LMDNAYFCEAD

Derivation of motif: Growth hormone receptor

**Supplementary Figure 1. Amino acid sequence of binding motifs.**

Juxtamembrane domain of c-KIT

TYKLQKPMYEVQWKVVEEINGNNFVFIDPTQLPYDHK

| JM-p | JM-b | JM-s | JM-z |
|---|---|---|---|
| 544–552 | 553–559 | 560–571 | 572–581 |

c-KIT-7YF

TYKLQKPMYEVQWKVVEEINGNNFVFIDPTQLPYDHKWEFPRNRLSFGKTLGAGAFGKVV
EATAYGLIKSDAAMTVAVKMLKPSAHLTEREALMSELKVLSYLGNHMNIVNLLGACTIGGP
TLVITEYCCYGDLLNFLRRKRDSFICSKQEDHAEAALFKNLLHSKESSCSDSTNEFMDMKPG
VSFVVPTKADKRRSVRIGSFIERDVTPAIMEDDELALDLEDLLSFSYQVAKGMAFLASKNCIH
RDLAARNILLTHGRITKICDFGLARDIKNDSNYVVKGNARLPVKWMAPESIFNCVYTFESDV
WSYGIFLWELFSLGSSPYPGMPVDSKFYKMIKEGFRMLSPEHAPAEMYDIMKTCWDADPLK
RPTFKQIVQLIEKQISESTNHIFSNLANCSPNRQKPVVDHSVRINSVGSTASSSQPLLVHDDV

ΔJMp: Deletion of JMp domain

YEVQWKVVEEINGNNFVFIDPTQLPYDHKWEFPRNRLSFGKTLGAGAFGKVVEATAYGLIK
SDAAMTVAVKMLKPSAHLTEREALMSELKVLSYLGNHMNIVNLLGACTIGGPTLVITEYCC
YGDLLNFLRRKRDSFICSKQEDHAEAALFKNLLHSKESSCSDSTNEFMDMKPGVSFVVPTKA
DKRRSVRIGSFIERDVTPAIMEDDELALDLEDLLSFSYQVAKGMAFLASKNCIHRDLAARNIL
LTHGRITKICDFGLARDIKNDSNYVVKGNARLPVKWMAPESIFNCVYTFESDVWSYGIFLWE
LFSLGSSPYPGMPVDSKFYKMIKEGFRMLSPEHAPAEMYDIMKTCWDADPLKRPTFKQIVQ
LIEKQISESTNHIFSNLANCSPNRQKPVVDHSVRINSVGSTASSSQPLLVHDDV

ΔJMpb: Deletion of JMpb domain

VEEINGNNFVFIDPTQLPYDHKWEFPRNRLSFGKTLGAGAFGKVVEATAYGLIKSDAAMTV
AVKMLKPSAHLTEREALMSELKVLSYLGNHMNIVNLLGACTIGGPTLVITEYCCYGDLLNFL
RRKRDSFICSKQEDHAEAALFKNLLHSKESSCSDSTNEFMDMKPGVSFVVPTKADKRRSVRI

GSFIERDVTPAIMEDDELALDLEDLLSFSYQVAKGMAFLASKNCIHRDLAARNILLTHGRITK

ICDFGLARDIKNDSNYVVKGNARLPVKWMAPESIFNCVYTFESDVWSYGIFLWELFSLGSSP

YPGMPVDSKFYKMIKEGFRMLSPEHAPAEMYDIMKTCWDADPLKRPTFKQIVQLIEKQISES

TNHIFSNLANCSPNRQKPVVDHSVRINSVGSTASSSQPLLVHDDV

ΔJMpbs: Deletion of JMpbs domain

DPTQLPYDHKWEFPRNRLSFGKTLGAGAFGKVVEATAYGLIKSDAAMTVAVKMLKPSAHL

TEREALMSELKVLSYLGNHMNIVNLLGACTIGGPTLVITEYCCYGDLLNFLRRKRDSFICSK

QEDHAEAALFKNLLHSKESSCSDSTNEFMDMKPGVSFVVPTKADKRRSVRIGSFIERDVTPA

IMEDDELALDLEDLLSFSYQVAKGMAFLASKNCIHRDLAARNILLTHGRITKICDFGLARDIK

NDSNYVVKGNARLPVKWMAPESIFNCVYTFESDVWSYGIFLWELFSLGSSPYPGMPVDSKFY

KMIKEGFRMLSPEHAPAEMYDIMKTCWDADPLKRPTFKQIVQLIEKQISESTNHIFSNLANC

SPNRQKPVVDHSVRINSVGSTASSSQPLLVHDDV

ΔJMpbsz: Deletion of JMpbsz domain

WEFPRNRLSFGKTLGAGAFGKVVEATAYGLIKSDAAMTVAVKMLKPSAHLTEREALMSELK

VLSYLGNHMNIVNLLGACTIGGPTLVITEYCCYGDLLNFLRRKRDSFICSKQEDHAEAALFK

NLLHSKESSCSDSTNEFMDMKPGVSFVVPTKADKRRSVRIGSFIERDVTPAIMEDDELALDL

EDLLSFSYQVAKGMAFLASKNCIHRDLAARNILLTHGRITKICDFGLARDIKNDSNYVVKGN

ARLPVKWMAPESIFNCVYTFESDVWSYGIFLWELFSLGSSPYPGMPVDSKFYKMIKEGFRML

SPEHAPAEMYDIMKTCWDADPLKRPTFKQIVQLIEKQISESTNHIFSNLANCSPNRQKPVVD

HSVRINSVGSTASSSQPLLVHDDV

**Supplementary Figure 2. Amino acid sequence of engineered c-KIT with a series of juxtamembrane deletions**

Red; tyrosine-to-phenylalanine mutation

pMK-Myr-F36V-(G4S)3-binding motif-ΔJMpbs c-KIT-myc-IRES-Puro$^{R}$-T2A-EGFP

- Myristoylation signal
- FKBP (F36V)
- $(GGGGS)_3$ linker
- binding motif
- ΔJMpbs c-KIT
- myc tag

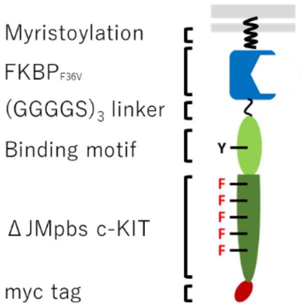


MGSSKSKPKDPSQRGSGGVQVETISPGDGRTFPKRGQTCVVHYTGMLEDGKKVDSSRDRN
KPFKFMLGKQEVIRGWEEGVAQMSVGQRAKLTISPDYAYGATGHPGIIPPHATLVFDVELL
KLEGSGGGGGSGGGGSGGGGSGRVbinding_motifDPTQLPYDHKWEFPRNRLSFGKTLGA
GAFGKVVEATAYGLIKSDAAMTVAVKMLKPSAHLTEREALMSELKVLSYLGNHMNIVNLLG
ACTIGGPTLVITEYCCYGDLLNFLRRKRDSFICSKQEDHAEAALFKNLLHSKESSCSDSTNEF
MDMKPGVSFVVPTKADKRRSVRIGSFIERDVTPAIMEDDELALDLEDLLSFSYQVAKGMAFL
ASKNCIHRDLAARNILLTHGRITKICDFGLARDIKNDSNYVVKGNARLPVKWMAPESIFNCV
YTFESDVWSYGIFLWELFSLGSSPYPGMPVDSKFYKMIKEGFRMLSPEHAPAEMYDIMKTC
WDADPLKRPTFKQIVQLIEKQISESTNHIFSNLANCSPNRQKPVVDHSVRINSVGSTASSSQP
LLVHDDVIDEQKLISEEDL*

**Supplementary Figure 3. Amino acid sequence of chemically dimerizable designer receptors.** Magenta; Myristoylation signal, Blue; FKBP (F36V), Gray; $(GGGGS)_3$ linker, Light green; binding motif, Dark green; ΔJMpbs c-KIT, Red; tyrosine-to-phenylalanine mutation, Brown; myc tag, Black; linker sequence

STAT3-binding motif

ΔJMpbs c-KIT

STAT3-binding motif at the N-terminus of engineered-c-KIT

VVHSGYRHQVPSDPTQLPYDHKWEFPRNRLSFGKTLGAGAFGKVVEATAYGLIKSDAAMT

VAVKMLKPSAHLTEREALMSELKVLSYLGNHMNIVNLLGACTIGGPTLVITEYCCYGDLLN

FLRRKRDSFICSKQEDHAEAALFKNLLHSKESSCSDSTNEFMDMKPGVSFVVPTKADKRRSV

RIGSFIERDVTPAIMEDDELALDLEDLLSFSYQVAKGMAFLASKNCIHRDLAARNILLTHGRI

TKICDFGLARDIKNDSNYVVKGNARLPVKWMAPESIFNCVYTFESDVWSYGIFLWELFSLGS

SPYPGMPVDSKFYKMIKEGFRMLSPEHAPAEMYDIMKTCWDADPLKRPTFKQIVQLIEKQIS

ESTNHIFSNLANCSPNRQKPVVDHSVRINSVGSTASSSQPLLVHDDV

STAT3-binding motif at Y703 of engineered-c-KIT

DPTQLPYDHKWEFPRNRLSFGKTLGAGAFGKVVEATAYGLIKSDAAMTVAVKMLKPSAHL

TEREALMSELKVLSYLGNHMNIVNLLGACTIGGPTLVITEYCCYGDLLNFLRRKRDSFICSK

QEDHVVHSGYRHQVPSKESSCSDSTNEFMDMKPGVSFVVPTKADKRRSVRIGSFIERDVTP

AIMEDDELALDLEDLLSFSYQVAKGMAFLASKNCIHRDLAARNILLTHGRITKICDFGLARDI

KNDSNYVVKGNARLPVKWMAPESIFNCVYTFESDVWSYGIFLWELFSLGSSPYPGMPVDSKF

YKMIKEGFRMLSPEHAPAEMYDIMKTCWDADPLKRPTFKQIVQLIEKQISESTNHIFSNLAN

CSPNRQKPVVDHSVRINSVGSTASSSQPLLVHDDV

STAT3-binding motif at Y721 of engineered-c-KIT

DPTQLPYDHKWEFPRNRLSFGKTLGAGAFGKVVEATAYGLIKSDAAMTVAVKMLKPSAHL

TEREALMSELKVLSYLGNHMNIVNLLGACTIGGPTLVITEYCCYGDLLNFLRRKRDSFICSK

QEDHAEAALFKNLLHSKESSCSVVHSGYRHQVPSVSFVVPTKADKRRSVRIGSFIERDVTPAI

MEDDELALDLEDLLSFSYQVAKGMAFLASKNCIHRDLAARNILLTHGRITKICDFGLARDIK

NDSNYVVKGNARLPVKWMAPESIFNCVYTFESDVWSYGIFLWELFSLGSSPYPGMPVDSKFY

KMIKEGFRMLSPEHAPAEMYDIMKTCWDADPLKRPTFKQIVQLIEKQISESTNHIFSNLANC

SPNRQKPVVDHSVRINSVGSTASSSQPLLVHDDV

STAT3-binding motif at Y730 of engineered-c-KIT

DPTQLPYDHKWEFPRNRLSFGKTLGAGAFGKVVEATAYGLIKSDAAMTVAVKMLKPSAHL

TEREALMSELKVLSYLGNHMNIVNLLGACTIGGPTLVITEYCCYGDLLNFLRRKRDSFICSK

QEDHAEAALFKNLLHSKESSCSDSTNEFMDMVVHSGYRHQVPSDKRRSVRIGSFIERDVTPA

IMEDDELALDLEDLLSFSYQVAKGMAFLASKNCIHRDLAARNILLTHGRITKICDFGLARDIK

NDSNYVVKGNARLPVKWMAPESIFNCVYTFESDVWSYGIFLWELFSLGSSPYPGMPVDSKFY

KMIKEGFRMLSPEHAPAEMYDIMKTCWDADPLKRPTFKQIVQLIEKQISESTNHIFSNLANC

SPNRQKPVVDHSVRINSVGSTASSSQPLLVHDDV

STAT3-binding motif at Y747 of engineered-c-KIT

DPTQLPYDHKWEFPRNRLSFGKTLGAGAFGKVVEATAYGLIKSDAAMTVAVKMLKPSAHL

TEREALMSELKVLSYLGNHMNIVNLLGACTIGGPTLVITEYCCYGDLLNFLRRKRDSFICSK

QEDHAEAALFKNLLHSKESSCSDSTNEFMDMKPGVSFVVPTKADKRRSVVHSGYRHQVPSP

AIMEDDELALDLEDLLSFSYQVAKGMAFLASKNCIHRDLAARNILLTHGRITKICDFGLARDI

KNDSNYVVKGNARLPVKWMAPESIFNCVYTFESDVWSYGIFLWELFSLGSSPYPGMPVDSKF

YKMIKEGFRMLSPEHAPAEMYDIMKTCWDADPLKRPTFKQIVQLIEKQISESTNHIFSNLAN

CSPNRQKPVVDHSVRINSVGSTASSSQPLLVHDDV

STAT3-binding motif at Y936 of engineered-c-KIT

DPTQLPYDHKWEFPRNRLSFGKTLGAGAFGKVVEATAYGLIKSDAAMTVAVKMLKPSAHL

TEREALMSELKVLSYLGNHMNIVNLLGACTIGGPTLVITEYCCYGDLLNFLRRKRDSFICSK

QEDHAEAALFKNLLHSKESSCSDSTNEFMDMKPGVSFVVPTKADKRRSVRIGSFIERDVTPA

IMEDDELALDLEDLLSFSYQVAKGMAFLASKNCIHRDLAARNILLTHGRITKICDFGLARDIK

NDSNYVVKGNARLPVKWMAPESIFNCVYTFESDVWSYGIFLWELFSLGSSPYPGMPVDSKFY

KMIKEGFRMLSPEHAPAEMYDIMKTCWDADPLKRPTFKQIVQLIEKQISEVVHSGYRHQVP

SSPNRQKPVVDHSVRINSVGSTASSSQPLLVHDDV

STAT3-binding motif at the C-terminus of engineered-c-KIT

DPTQLPYDHKWEFPRNRLSFGKTLGAGAFGKVVEATAYGLIKSDAAMTVAVKMLKPSAHL

TEREALMSELKVLSYLGNHMNIVNLLGACTIGGPTLVITEYCCYGDLLNFLRRKRDSFICSK

QEDHAEAALFKNLLHSKESSCSDSTNEFMDMKPGVSFVVPTKADKRRSVRIGSFIERDVTPA

IMEDDELALDLEDLLSFSYQVAKGMAFLASKNCIHRDLAARNILLTHGRITKICDFGLARDIK

NDSNYVVKGNARLPVKWMAPESIFNCVYTFESDVWSYGIFLWELFSLGSSPYPGMPVDSKFY

KMIKEGFRMLSPEHAPAEMYDIMKTCWDADPLKRPTFKQIVQLIEKQISESTNHIFSNLANC

SPNRQKPVVDHSVRINSVGSTASSSQPLLVHDDVVVHSGYRHQVPS

**Supplementary Figure 4. Amino acid sequence of engineered c-KIT variants with differential positions of the STAT3-binding motif.** Light green; STAT3-binding motif, Dark Green; ΔJMpbs c-KIT, Red; tyrosine-to-phenylalanine mutation

pMK-Myr-F36V-(G4S)3-binding motif-ΔJMpbs c-KIT-binding motif-myc-IRES-Puro$^{R}$-T2A-EGFP

- Myristoylation signal
- FKBP (F36V)
- (GGGGS)$_3$ linker
- binding motif 1
- ΔJMpbs c-KIT
- binding motif 2
- myc tag

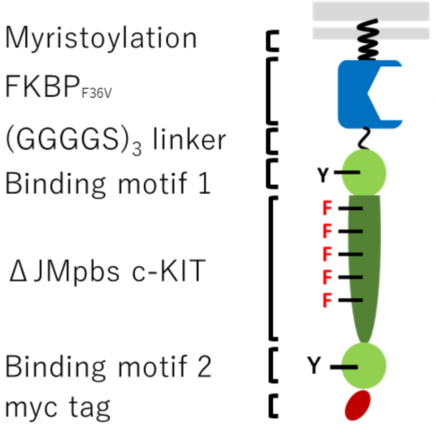


MGSSKSKPKDPSQRGSGGVQVETISPGDGRTFPKRGQTCVVHYTGMLEDGKKVDSSRDRN

KPFKFMLGKQEVIRGWEEGVAQMSVGQRAKLTISPDYAYGATGHPGIIPPHATLVFDVELL

KLEGSGGGGGSGGGGSGGGGSGRVBinding_motif1DPTQLPYDHKWEFPRNRLSFGKTLG

AGAFGKVVEATAYGLIKSDAAMTVAVKMLKPSAHLTEREALMSELKVLSYLGNHMNIVNLL

GACTIGGPTLVITEYCCYGDLLNFLRRKRDSFICSKQEDHAEAALFKNLLHSKESSCSDSTNE

FMDMKPGVSFVVPTKADKRRSVRIGSFIERDVTPAIMEDDELALDLEDLLSFSYQVAKGMAF

LASKNCIHRDLAARNILLTHGRITKICDFGLARDIKNDSNYVVKGNARLPVKWMAPESIFNC

VYTFESDVWSYGIFLWELFSLGSSPYPGMPVDSKFYKMIKEGFRMLSPEHAPAEMYDIMKTC

WDADPLKRPTFKQIVQLIEKQISESTNHIFSNLANCSPNRQKPVVDHSVRINSVGSTASSSQP

LLVHDDVBinding_motif2IDEQKLISEEDL*

**Supplementary Figure 5. Amino acid sequence of designer receptors with two tyrosine motifs.** Magenta; Myristoylation signal, Blue; FKBP (F36V), Gray; (GGGGS)$_3$ linker, Light green; binding motifs 1 and 2, Dark green; ΔJMpbs c-KIT, Red; tyrosine-to-phenylalanine mutation, Brown; myc tag, Black; linker sequence

pMK-Myr-LOV-(G4S)3-ΔJMpbs c-KIT-STAT3 binding motif-myc-IRES-Puro$^{R}$-T2A-EGFP

Myristoylation signal
LOV domain
(GGGGS)$_3$ linker
ΔJMpbs c-KIT
STAT3-binding motif
myc tag

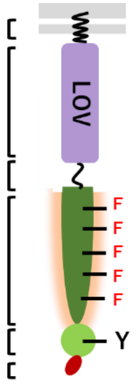


MGSSKSKPKDPSQRGSGPDYSLVKALQMAQQNFVITDASLPDNPIVYASRGFLTLTGYSLD
QILGRNCRFLQGPETDPRAVDKIRNAITKGVDTSVCLLNYRQDGTTFWNLFFVAGLRDSKG
NIVNYVGVQSKVSEDYAKLLVNEQNIEYKGVRTSNMLRRKGSGGGGGSGGGGSGGGGSGR
VDPTQLPYDHKWEFPRNRLSFGKTLGAGAFGKVVEATAYGLIKSDAAMTVAVKMLKPSAH
LTEREALMSELKVLSYLGNHMNIVNLLGACTIGGPTLVITEYCCYGDLLNFLRRKRDSFICSK
QEDHAEAALFKNLLHSKESSCSDSTNEFMDMKPGVSFVVPTKADKRRSVRIGSFIERDVTPA
IMEDDELALDLEDLLSFSYQVAKGMAFLASKNCIHRDLAARNILLTHGRITKICDFGLARDIK
NDSNYVVKGNARLPVKWMAPESIFNCVYTFESDVWSYGIFLWELFSLGSSPYPGMPVDSKFY
KMIKEGFRMLSPEHAPAEMYDIMKTCWDADPLKRPTFKQIVQLIEKQISESTNHIFSNLANC
SPNRQKPVVDHSVRINSVGSTASSSQPLLVHDDVVVHSGYRHQVPSIDEQKLISEEDL*

**Supplementary Figure 6. Amino acid sequence of blue light-responsive designer receptors.** Magenta; Myristoylation signal, Purple; LOV domain, Gray; (GGGGS)$_3$ linker, Light green; STAT3-binding motif, Dark green; ΔJMpbs c-KIT, Red; tyrosine-to-phenylalanine mutation, Brown; myc tag, Black; linker sequence

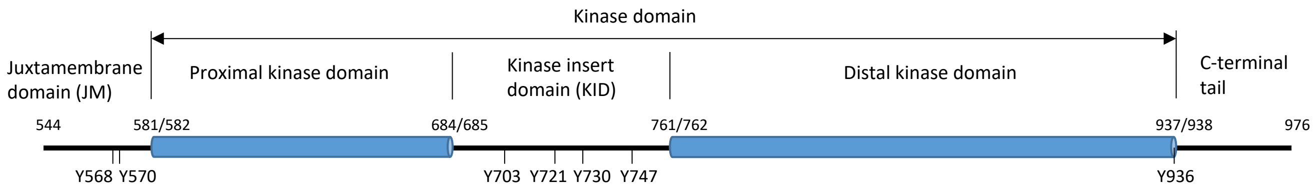


**Supplementary Figure 7. Domain structure of c-KIT intracellular domain**

The domain names, amino acid numbering, and engineered 7 tyrosine residues are indicated.

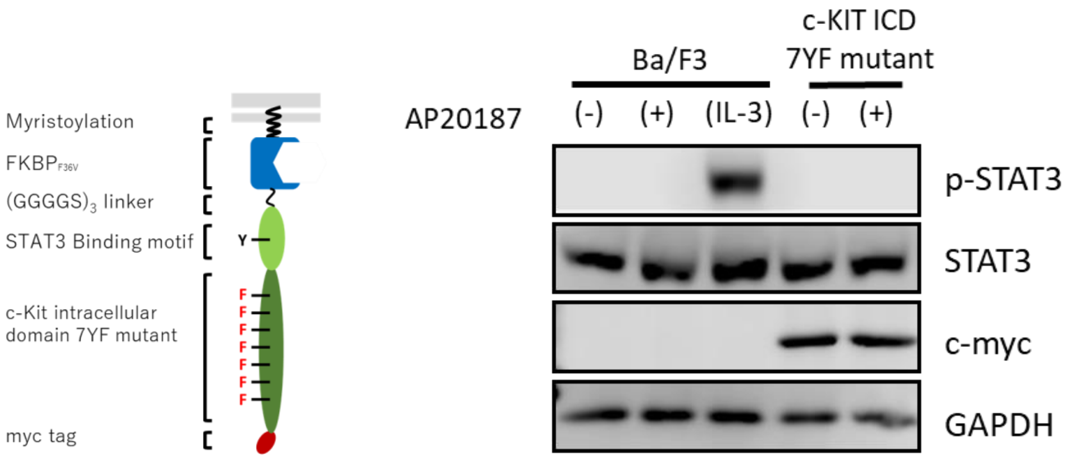


**Supplementary Figure 8. Detecting phosphorylation of STAT3 induced by the firstly constructed c-KIT 7YF-based designer receptor.** The intracellular domain of c-KIT (c-KIT ICD) received phenylalanine point mutations at seven tyrosine residues (7YF) which act as binding sites for signaling molecules. The c-KIT ICD/7YF domain was used as a kinase domain of the designer receptor.

In western blotting, parental Ba/F3 and the transduced cells were unstimulated (−) or stimulated with 50 nM AP20187(+) or 1 ng/ml IL-3. Phospho-STAT3, whole STAT3, c-myc tag (chimeric receptor expression), and GAPDH were detected using corresponding primary antibodies.

The full uncropped blot images are provided as Supplementary Fig. 17.

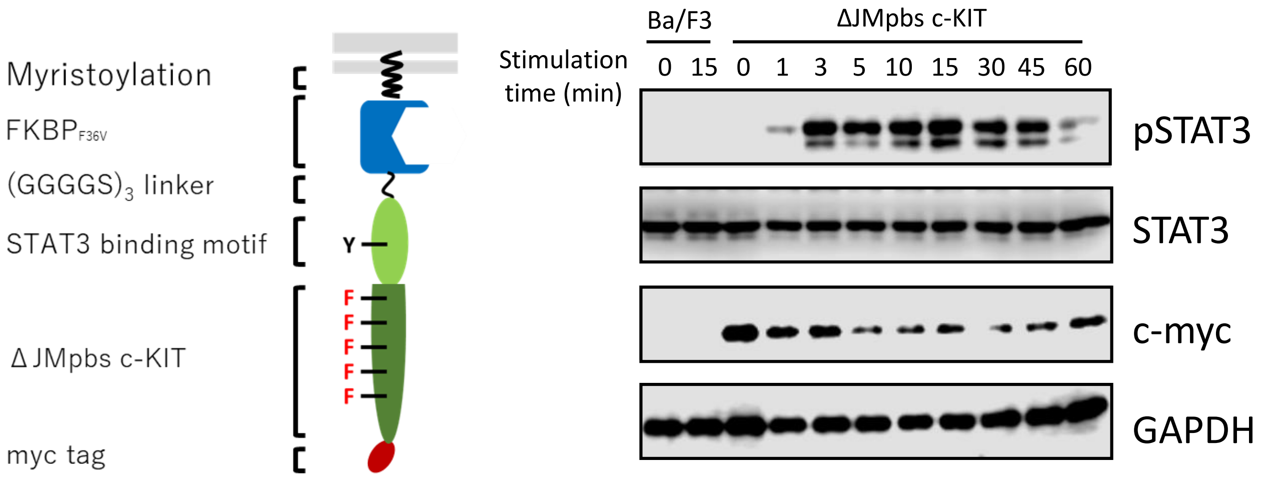


**Supplementary Figure 9. Time-dependent phosphorylation of STAT3 by a designer receptor.** The intracellular domain of c-KIT (c-KIT ICD) was engineered by deleting JM (position 544-571) and adding phenylalanine point mutations at five tyrosine residues which act as binding sites for downstream signaling mediators (Y703, Y721, Y730, Y823, and Y936) to yield ΔJMpbs c-KIT. The ΔJMpbs c-KIT was used as a kinase domain of the designer receptor. In western blotting, parental Ba/F3 and the transduced cells were unstimulated (0) or stimulated with 50 nM AP20187 for the indicated time periods. Phospho-STAT3, whole STAT3, c-myc tag (chimeric receptor expression), and GAPDH were detected using corresponding primary antibodies.

The full uncropped blot images are provided as Supplementary Fig. 17.

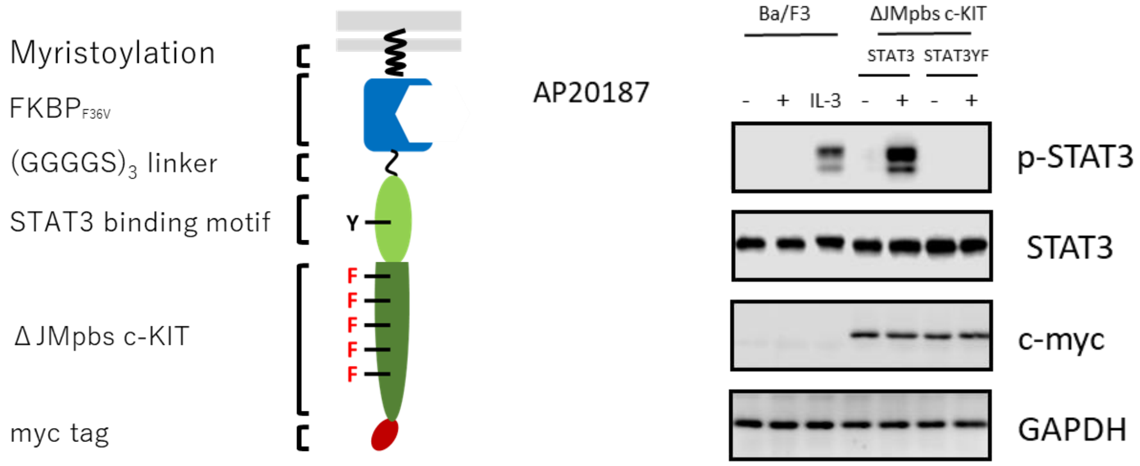


**Supplementary Figure 10. Phosphorylation of STAT3 depended on tyrosine phosphorylation of the binding motif.** Parental Ba/F3 and the transduced cells were unstimulated (−) or stimulated with 50 nM AP20187(+) or 1 ng/ml IL-3. As a negative control, the designer receptor which have the tyrosine to phenylalanine mutation of the STAT3-binding motif (STAT3YF) was created. Phospho-STAT3, whole STAT3, c-myc tag (chimeric receptor expression), and GAPDH were detected using corresponding primary antibodies.

The full uncropped blot images are provided as Supplementary Fig. 17.

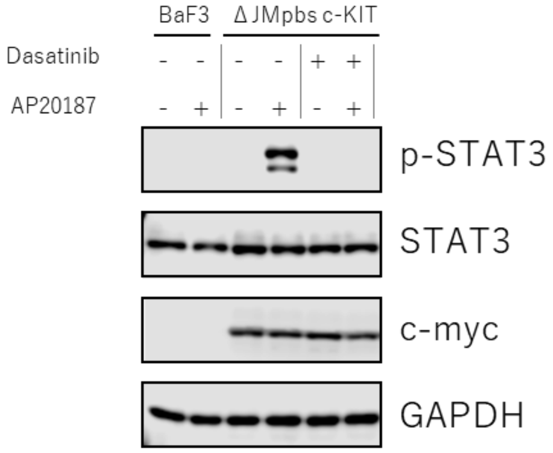


**Supplementary Figure 11. Phosphorylation of STAT3 depended on the engineered kinase domain.** Parental Ba/F3 and the transduced cells were unstimulated (−) or stimulated with 50 nM AP20187(+). To verify the tyrosine kinase dependence of STAT3 phosphorylation, the cells were treated by 1 µM Dasatinib. Phospho-STAT3, whole STAT3, c-myc tag (chimeric receptor expression), and GAPDH were detected using corresponding primary antibodies.

The full uncropped blot images are provided as Supplementary Fig. 17.

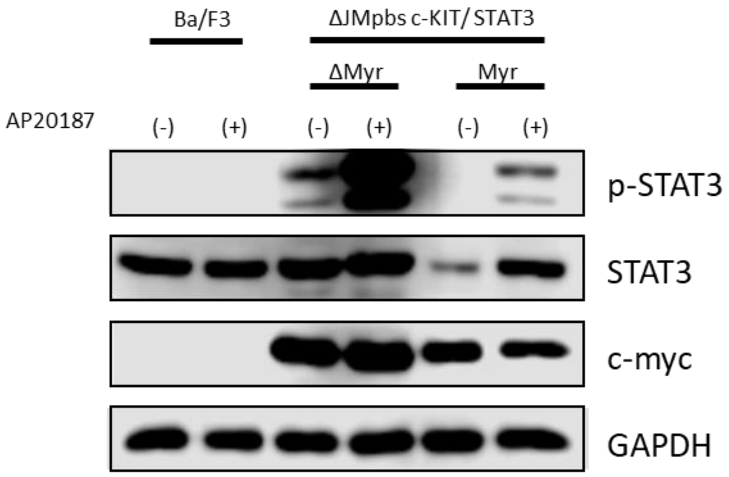


**Supplementary Figure 12. Myristoylation affected the kinase activity level of the designer receptor.** Myristoylation was deleted from the designer receptor (ΔMyr) to investigate the effect of membrane localization. In western blotting, parental Ba/F3 and the transduced cells were unstimulated (−) or stimulated with 50 nM AP20187(+). Phospho-STAT3, whole STAT3, c-myc tag (chimeric receptor expression), and GAPDH were detected using corresponding primary antibodies.

The full uncropped blot images are provided as Supplementary Fig. 17.

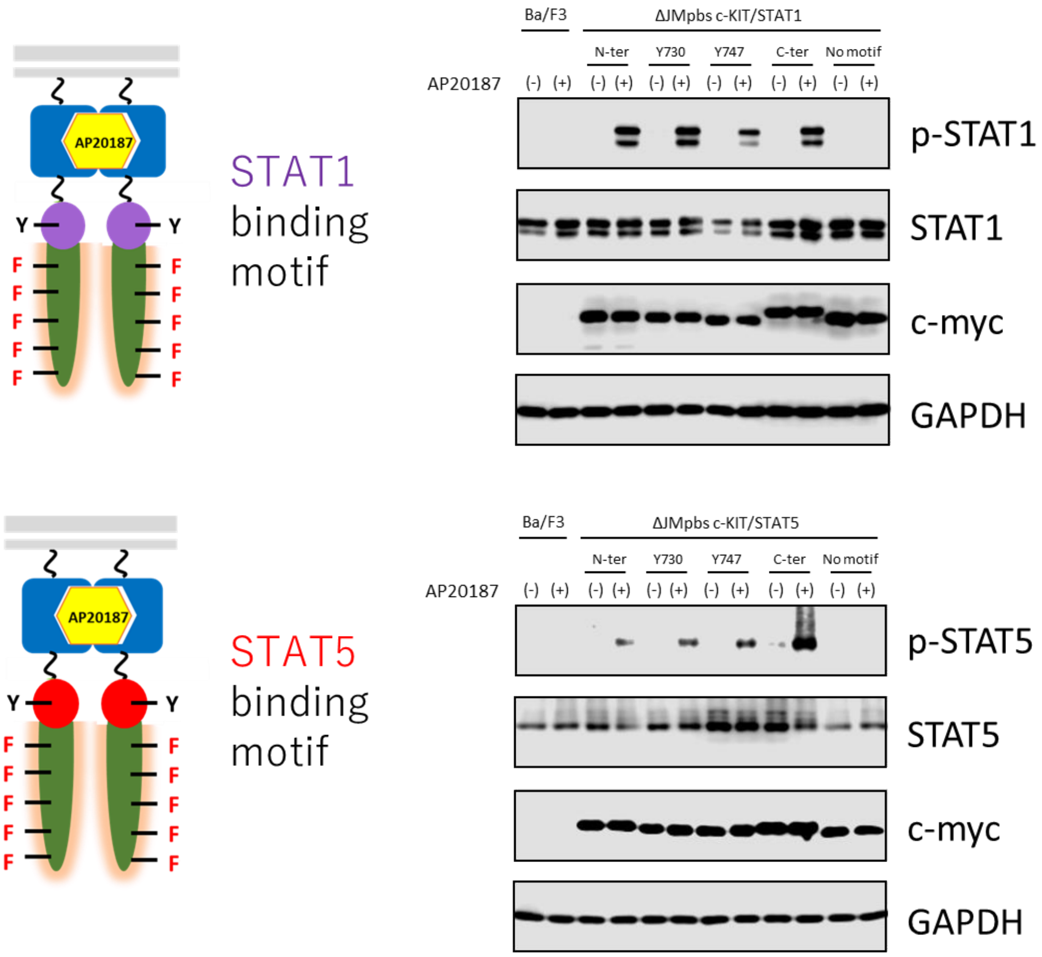


**Supplementary Figure 13. Exploring appropriate positions of tyrosine motifs located in the engineered c-KIT.** Similar to the constructs using the STAT3-binding motif (Fig. 3A), the receptors which have the STAT1- or STAT5-binding motif were constructed. The construct in which the binding motif was deleted (no motif) was used as a negative control. In western blotting, parental Ba/F3 and the transduced cells were unstimulated (−) or stimulated with 50 nM AP20187(+). Phospho-STAT1, whole STAT1, Phospho-STAT3, whole STAT3, Phospho-STAT5, whole STAT5, c-myc tag (chimeric receptor expression), and GAPDH were detected using corresponding primary antibodies.

The full uncropped blot images are provided as Supplementary Fig. 17.

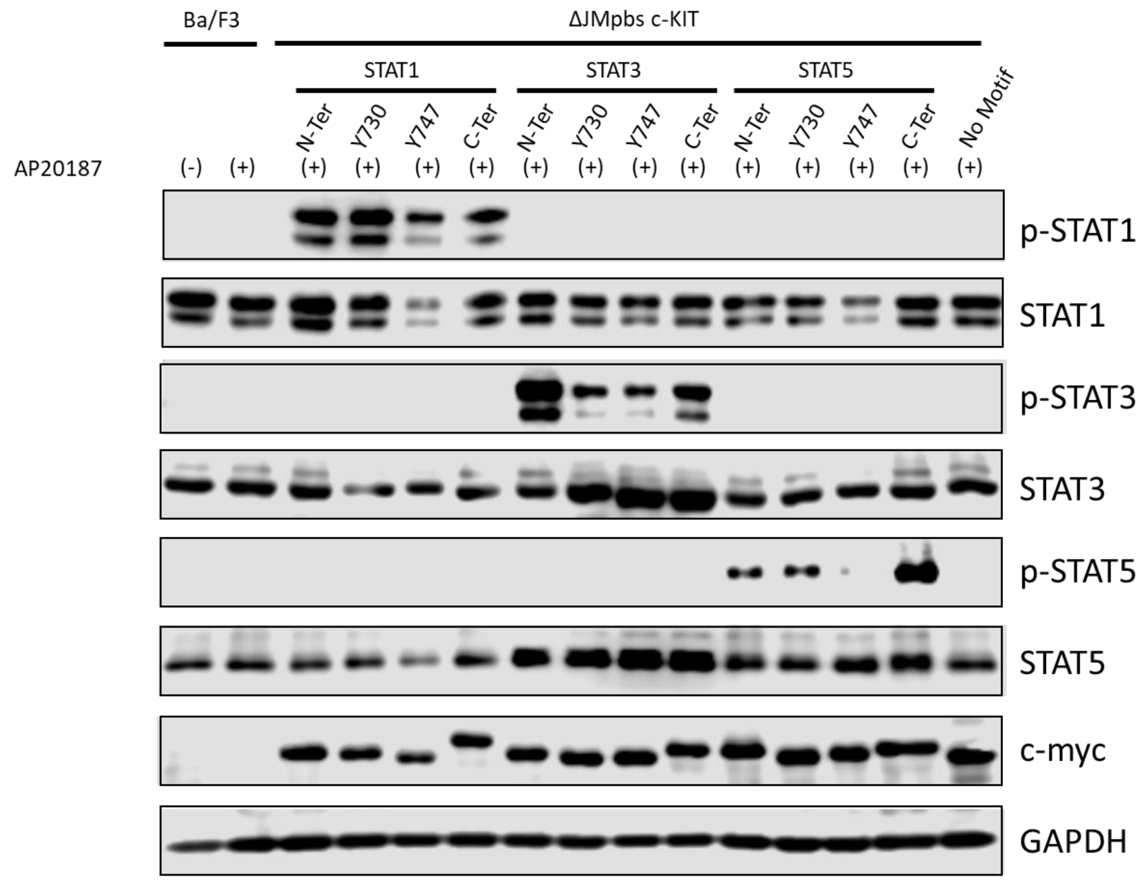


**Supplementary Figure 14. Comparison of the phosphorylation levels of signaling molecules induced by designer receptors with differential tyrosine motif positions.** Four positions on engineered c-KIT, *i.e.* the N-terminus, Y730, Y747, and the C-terminus, are replaced by the corresponding motif. In western blotting, parental Ba/F3 and the transduced cells were stimulated with 50 nM AP20187(+). Phospho-STAT1, whole STAT1, Phospho-STAT3, whole STAT3, Phospho-STAT5, whole STAT5, c-myc tag (chimeric receptor expression), and GAPDH were detected using corresponding primary antibodies.

The full uncropped blot images are provided as Supplementary Fig. 17.

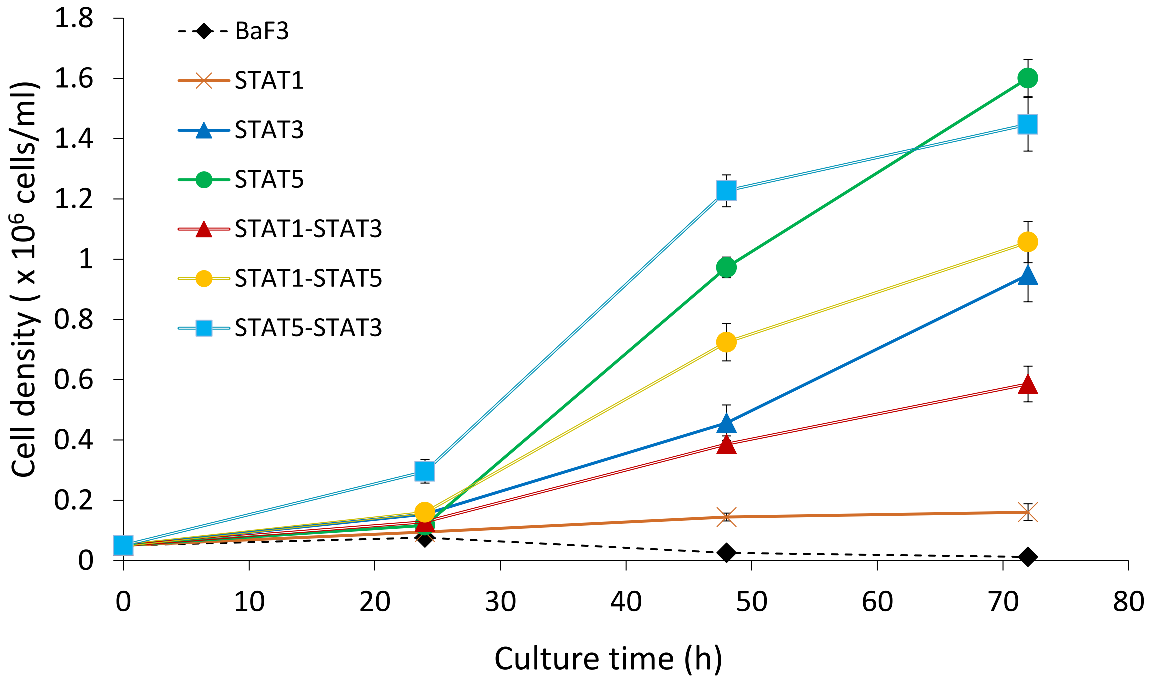


**Supplementary Figure 15. Cell proliferation properties of designer receptor-expressing cells.**

A cell proliferation assay was performed for the Ba/F3 transductants expressing the designer receptors with either STAT1, STAT3, or STAT5-binding motif at the N-terminus, and those with two binding motifs in combination of either STAT1-STAT3, STAT1-STAT5, and STAT5-STAT3 at N- and C-termini. Cells were seeded into 24-well plates at day 0 ($5 \times 10^4$ cells/ml) and cultured in the medium with 50 nM AP20187. At the indicated time points, cell number was counted by flow cytometry. Viable cell densities in triplicate cultures are plotted as mean ± SD.

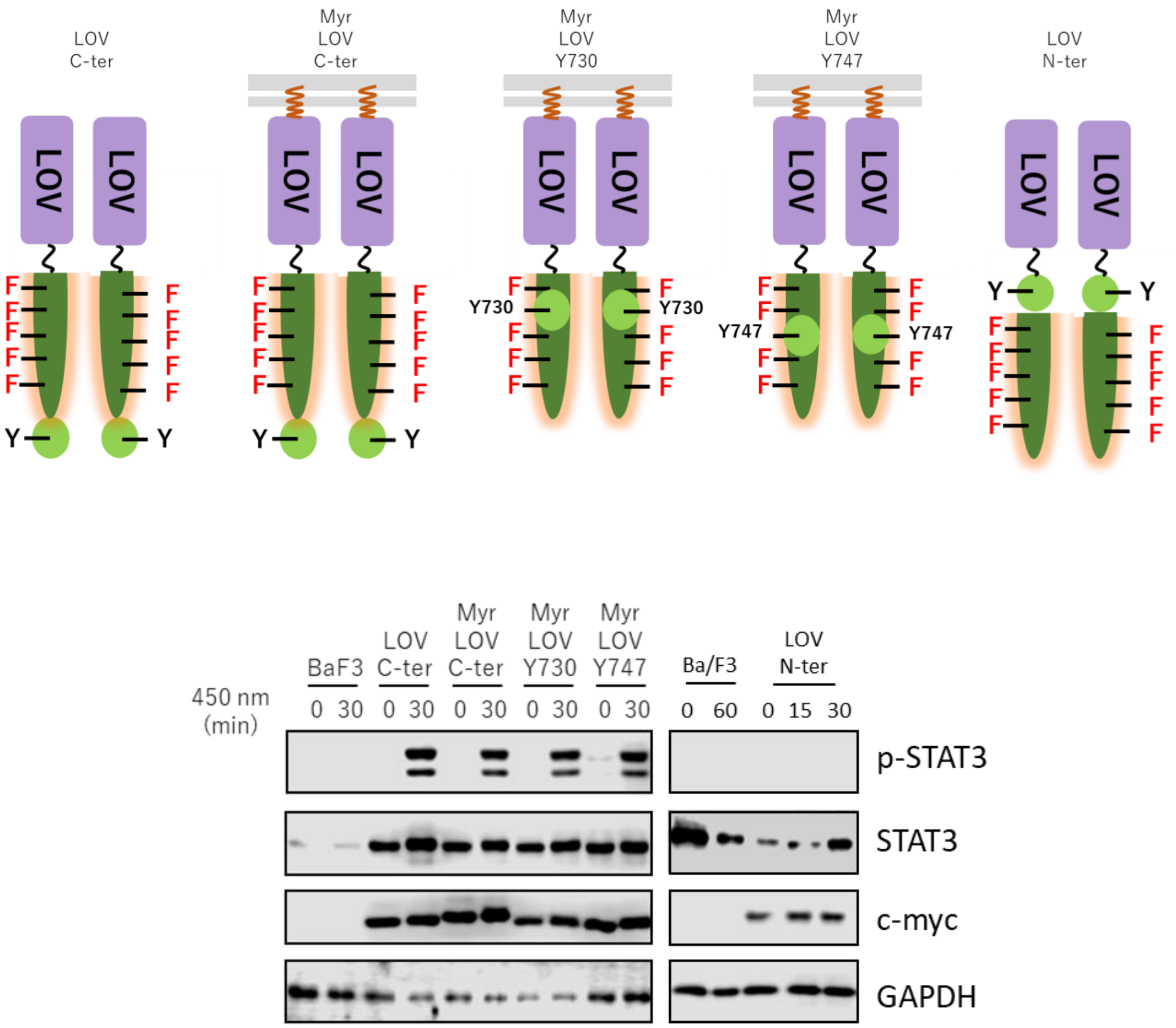


**Supplementary Figure 16. Light-inducible designer receptors with differential tyrosine motif positions.** The designer receptors whose tyrosine motif was added at the N-terminus, or C-terminus, or replaced at Y730 or Y747 of the engineered kinase domain were designed. In western blotting, parental Ba/F3 and the transduced cells were unstimulated (0) or stimulated for 30 min with blue light (0.5 mW/cm$^2$, 450 nm). Phospho-STAT3, whole STAT3, c-myc tag (chimeric receptor expression), and GAPDH were detected using corresponding primary antibodies.

The full uncropped blot images are provided as Supplementary Fig. 17.

**Supplementary Figure 17. The full uncropped blot images.**

Main Figure 2B

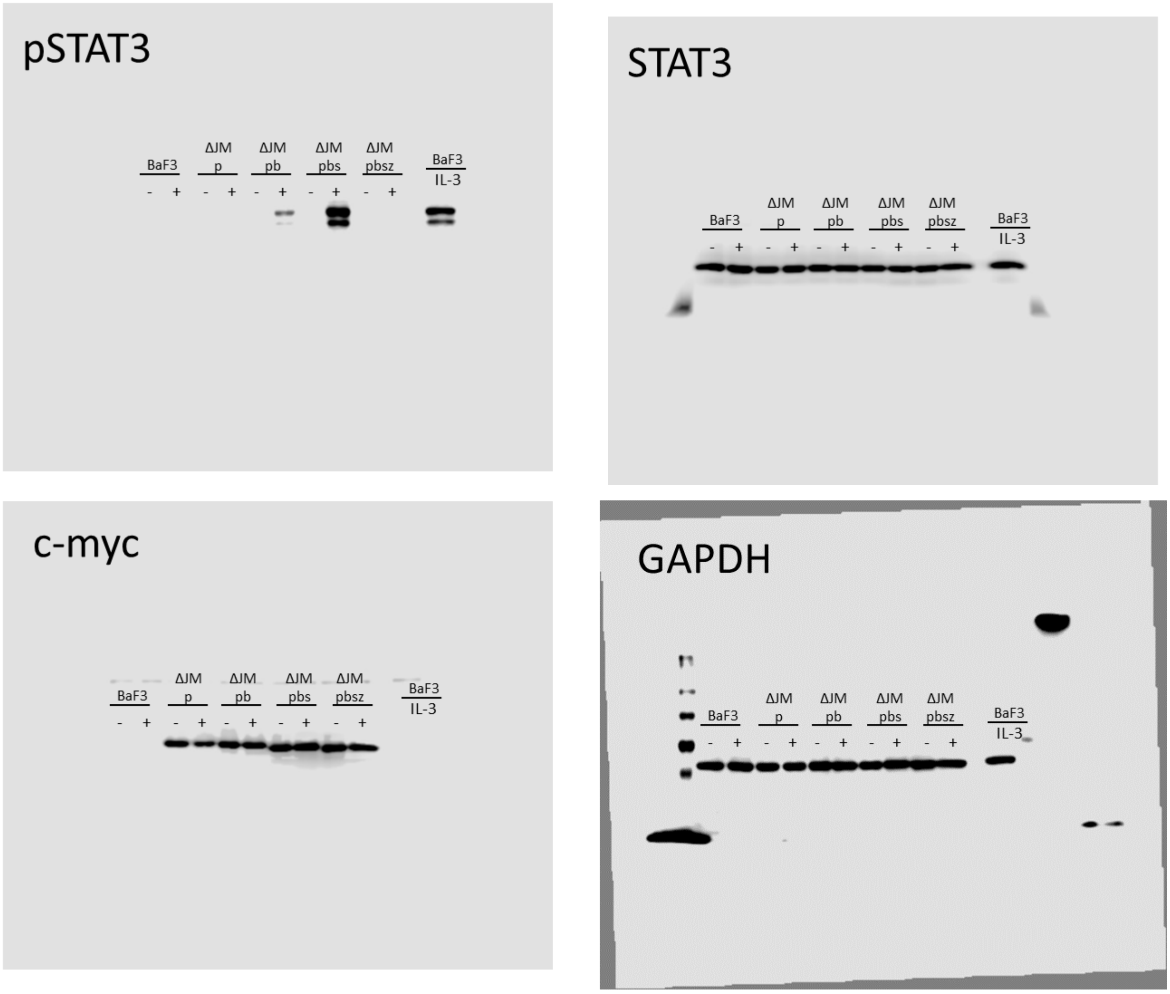

Main Figure 2C

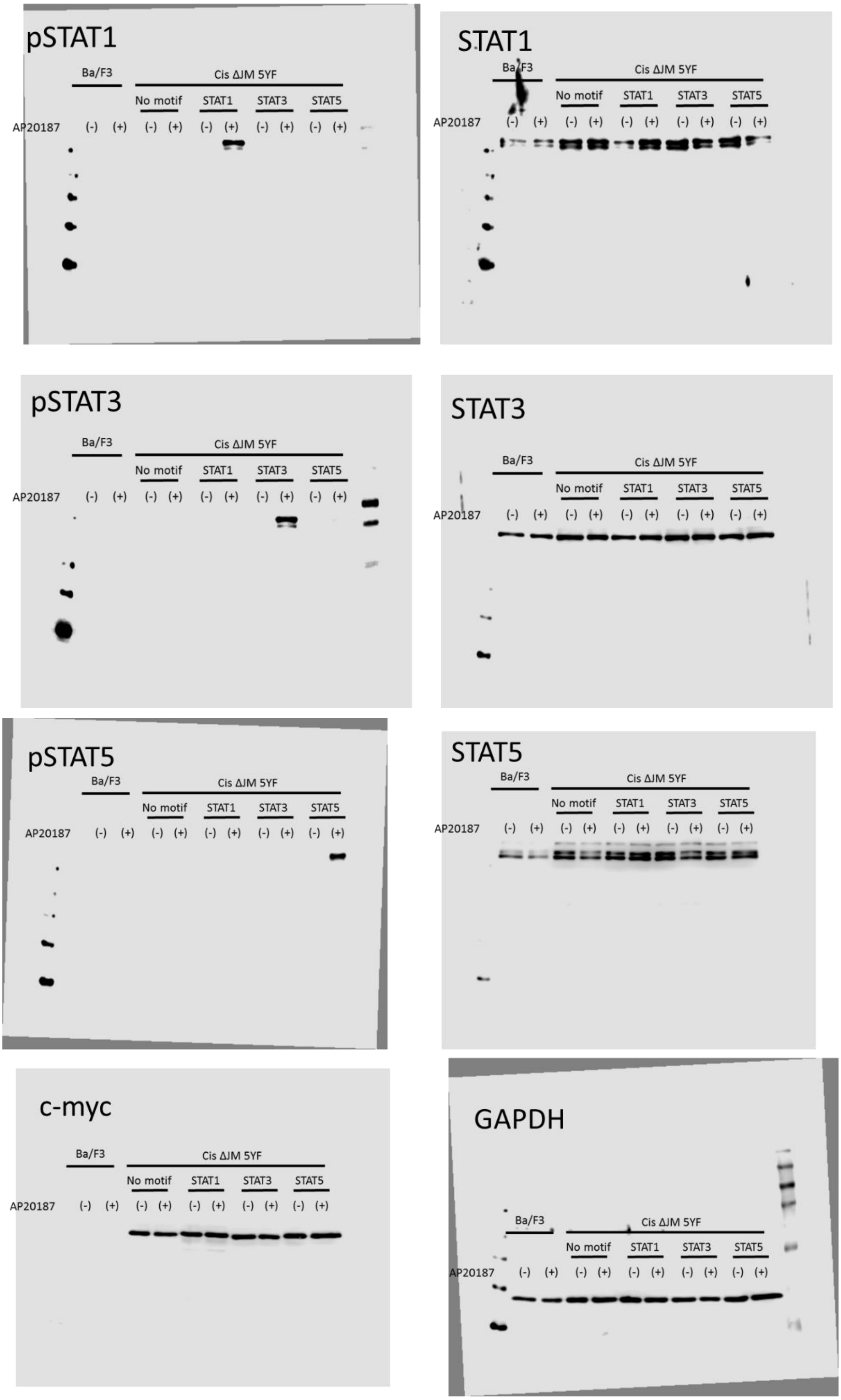

Main Figure 3A

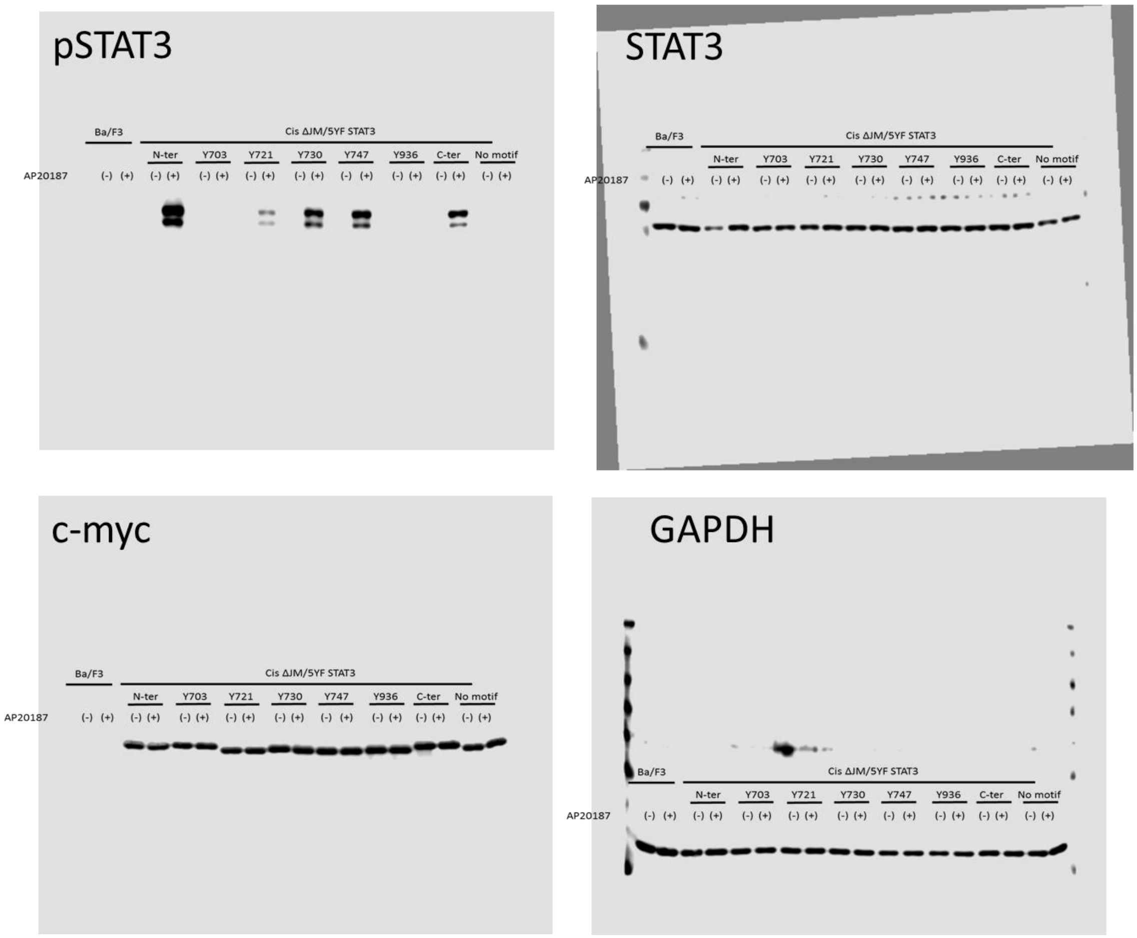

Main Figure 3B

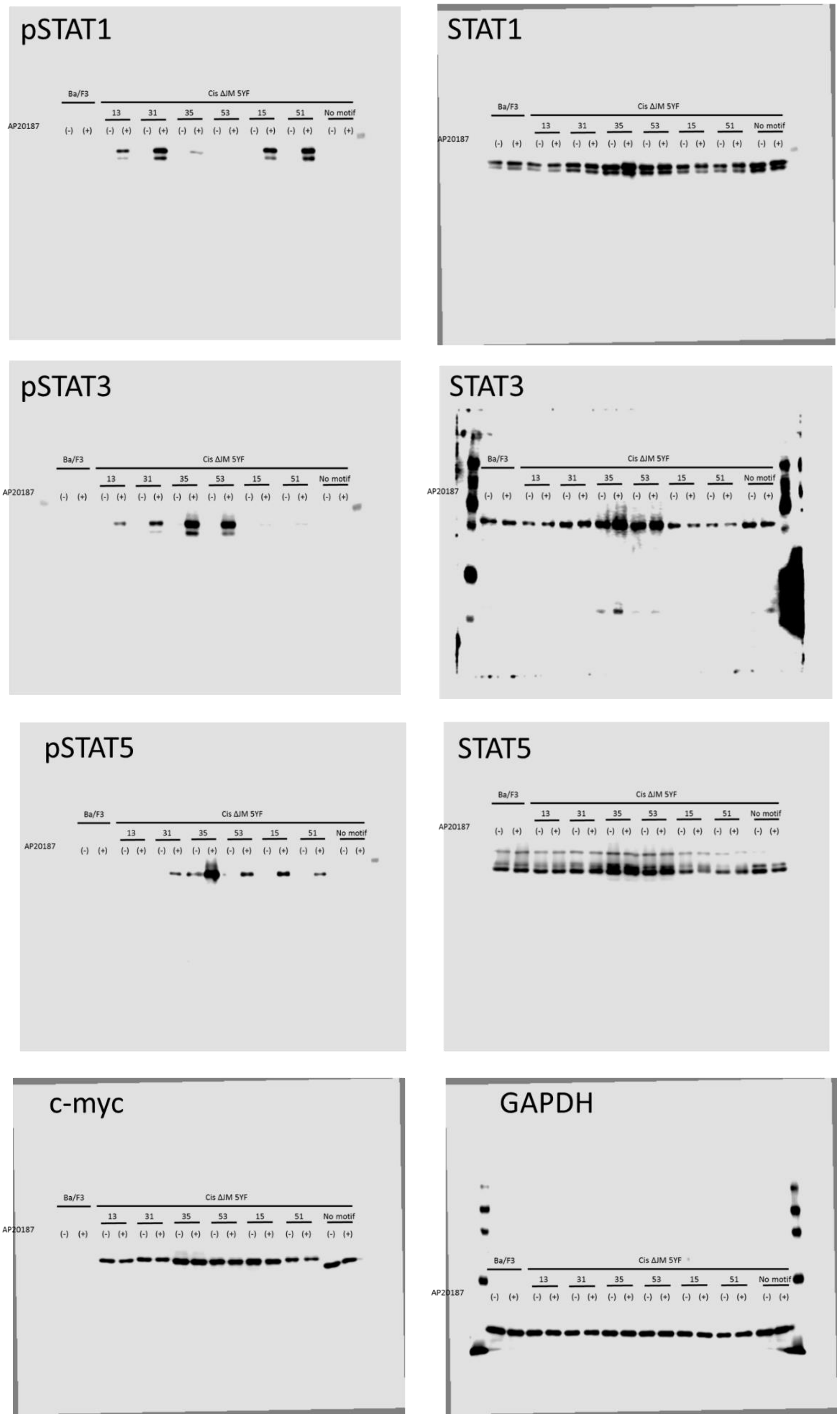

Main Figure 4A

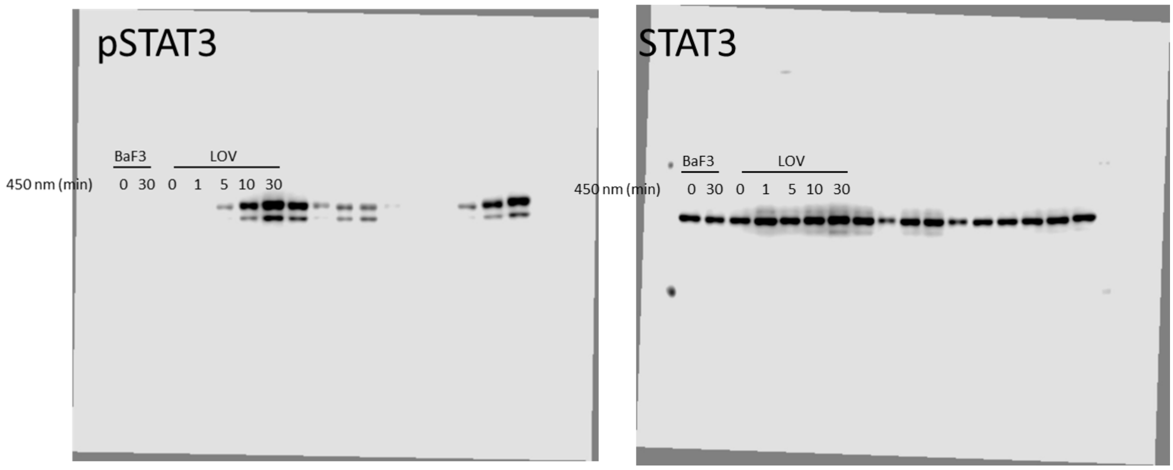


Main Figure 4B

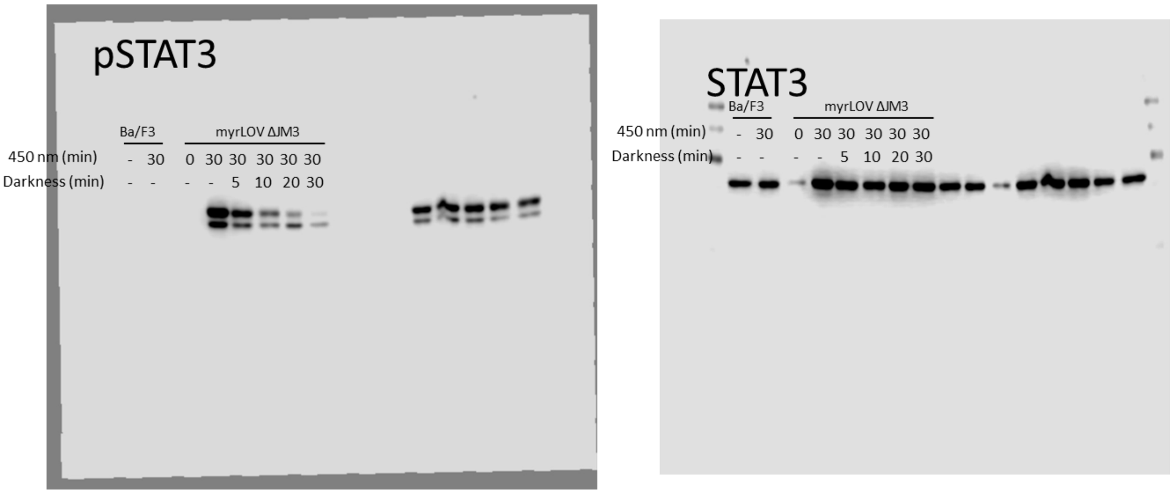


Main Figure 4C

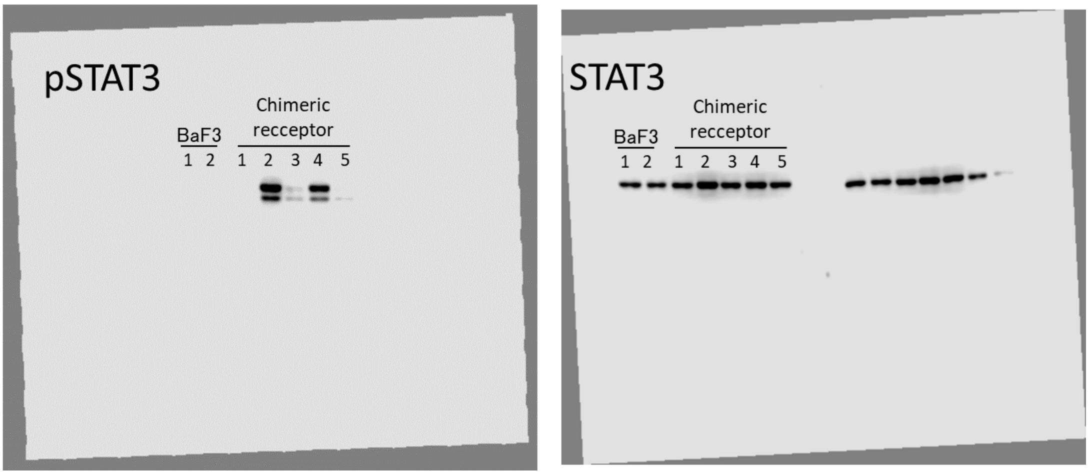

Supplementary Figure 8

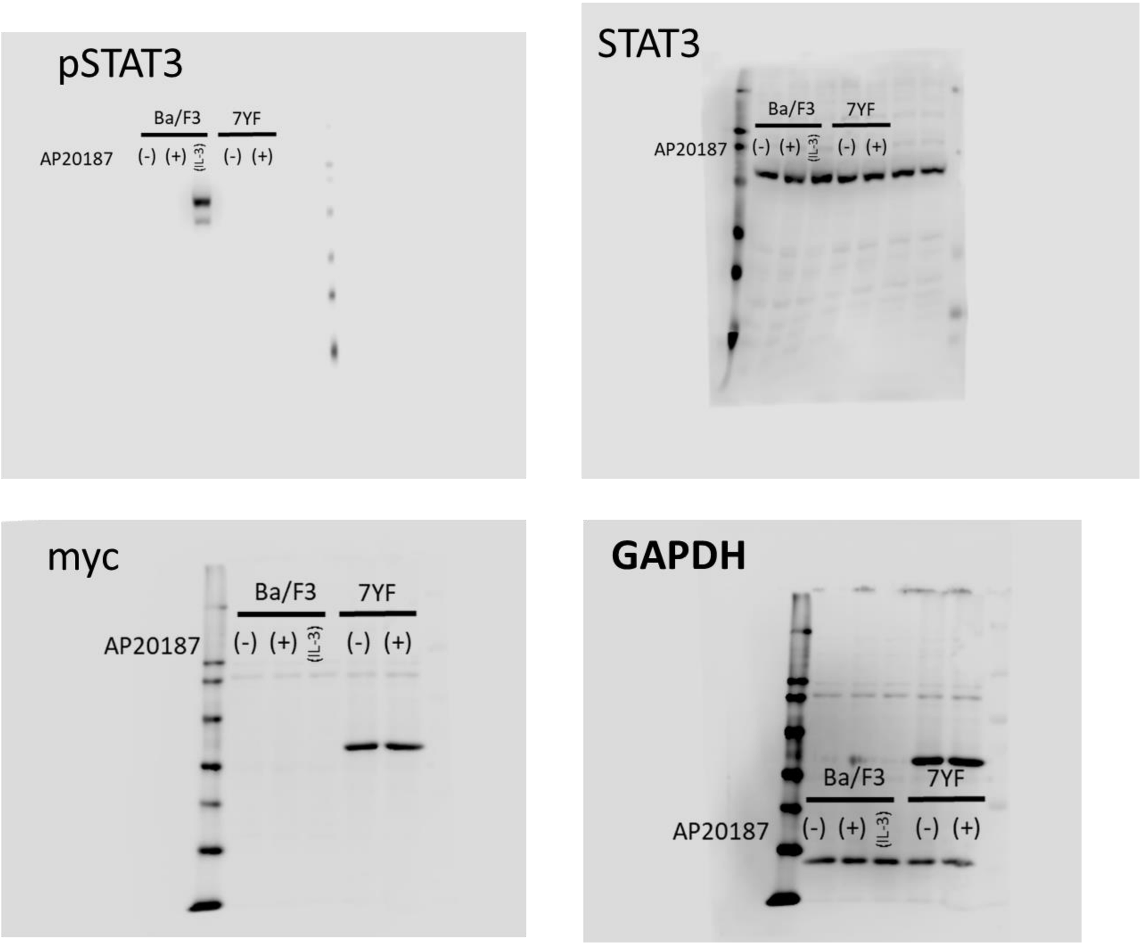

Supplementary Figure 9

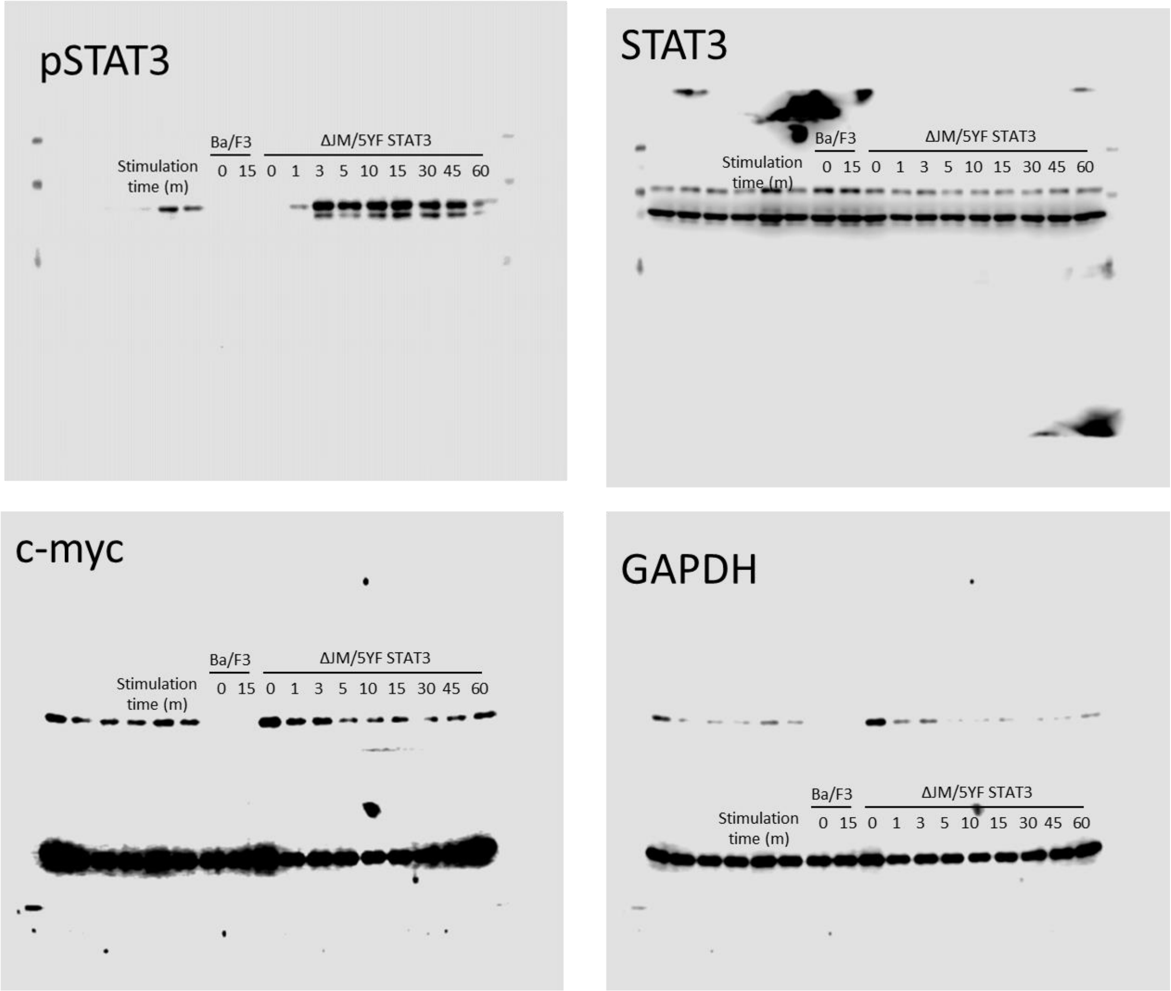

Supplementary Figure 10

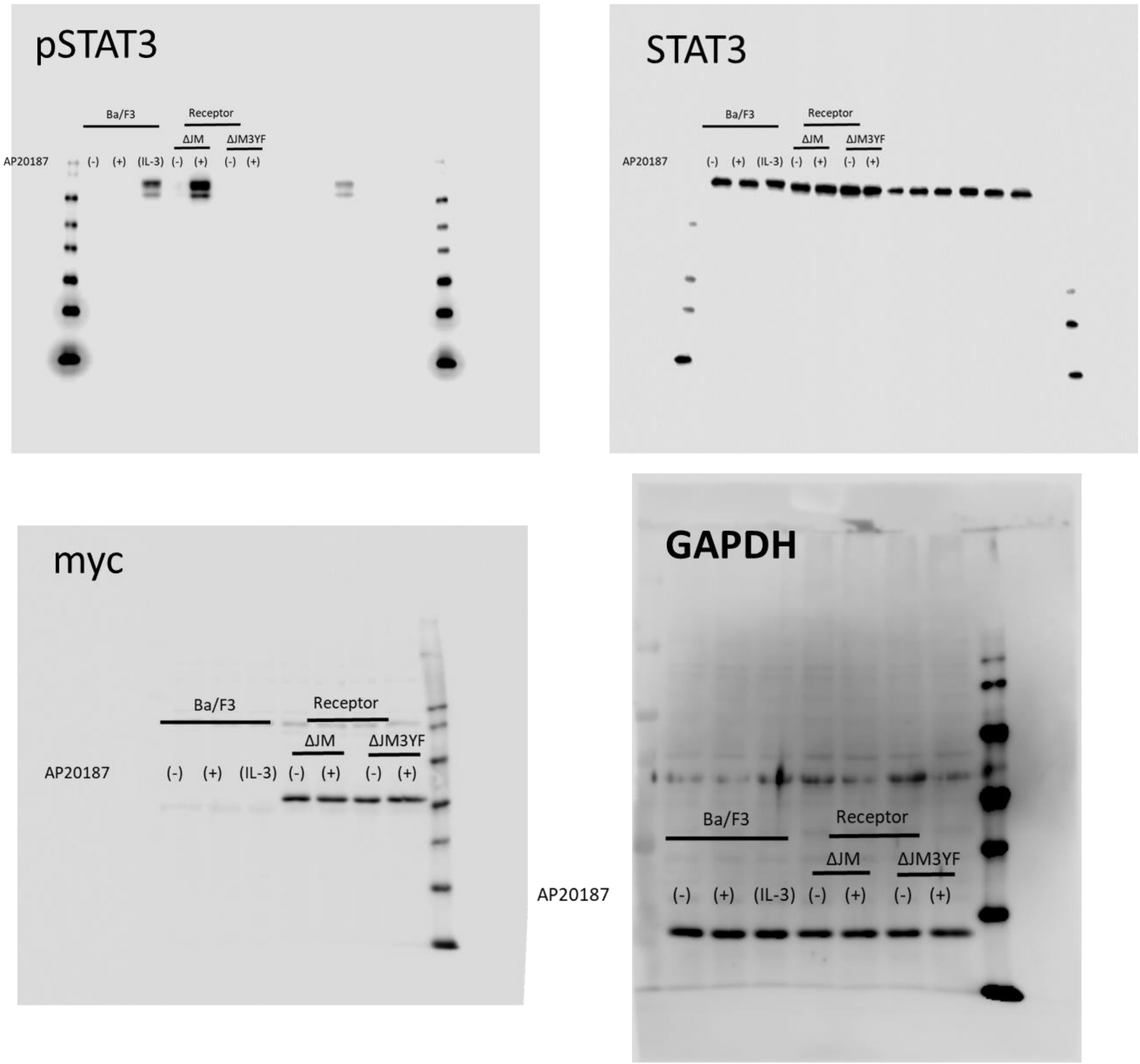

Supplementary Figure 11

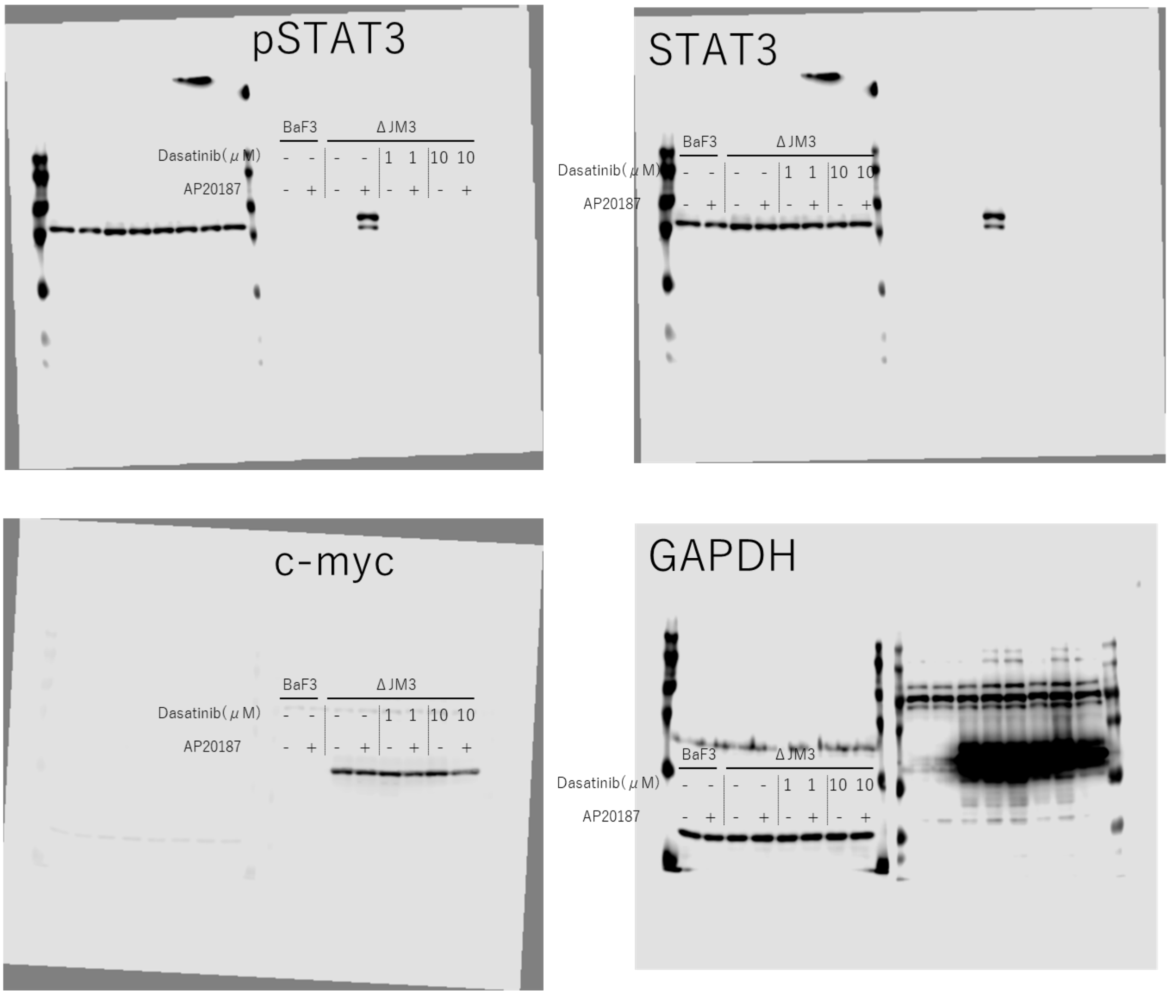

Supplementary Figure 12

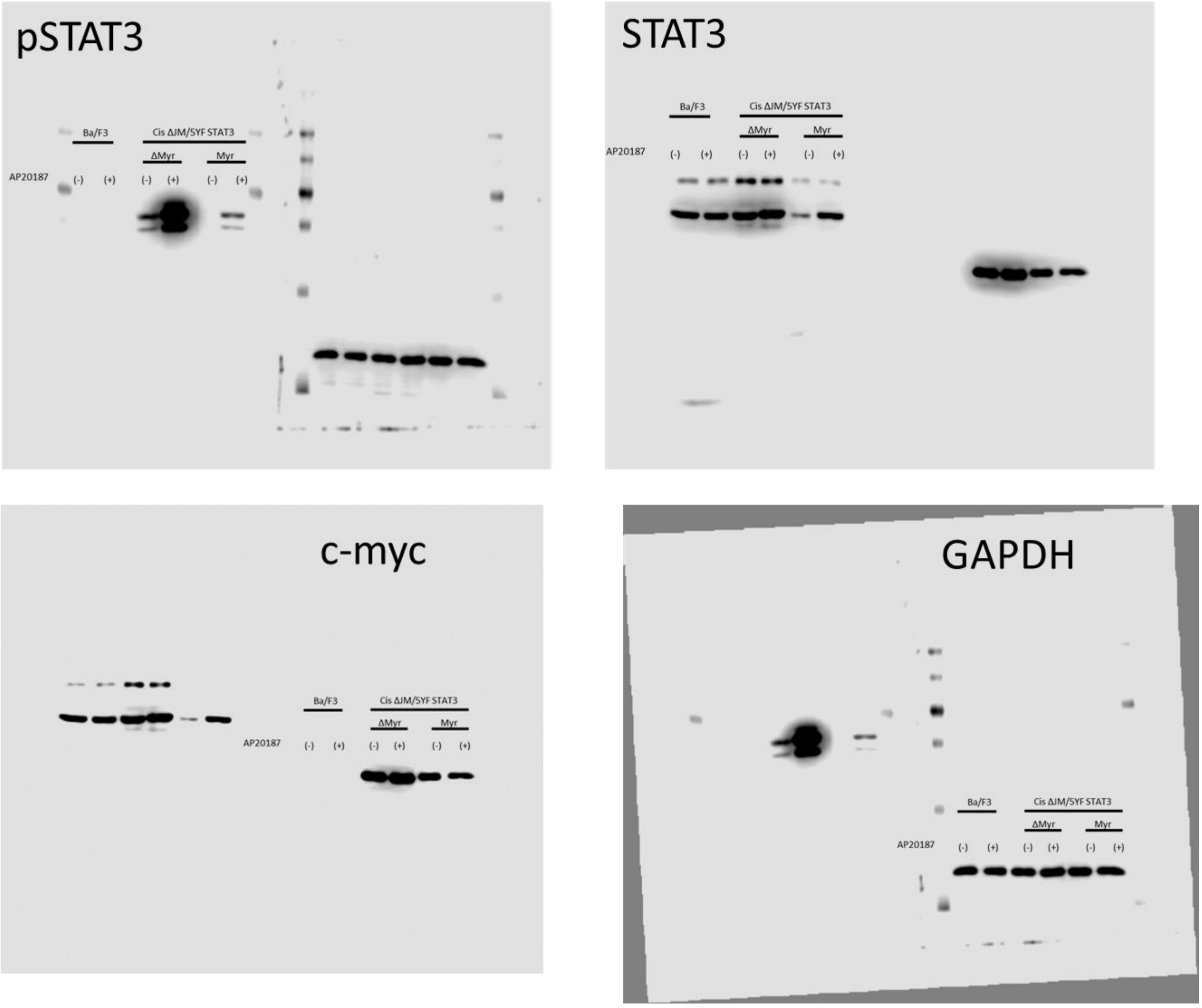

Supplementary Figure 13

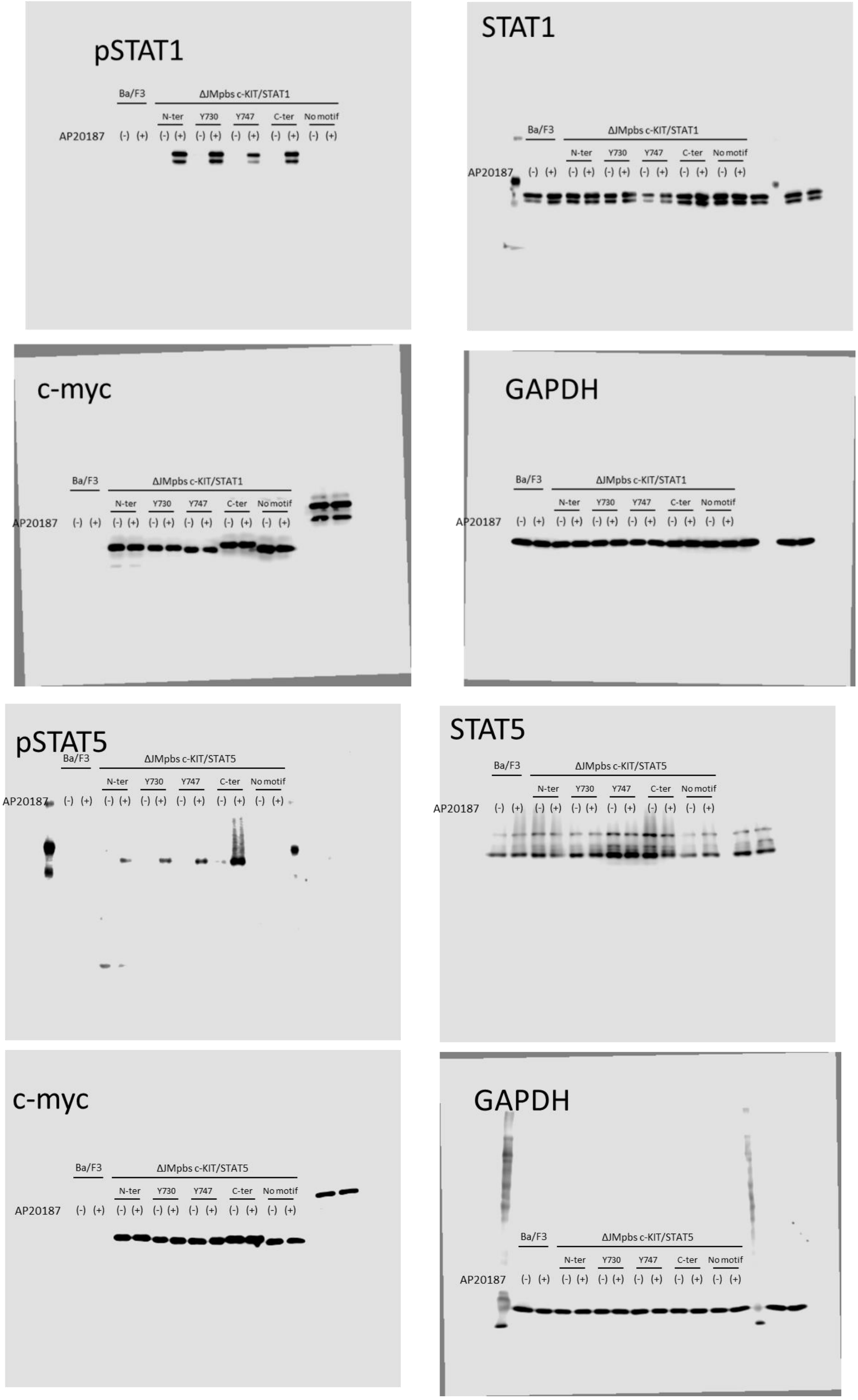

Supplementary Figure 14

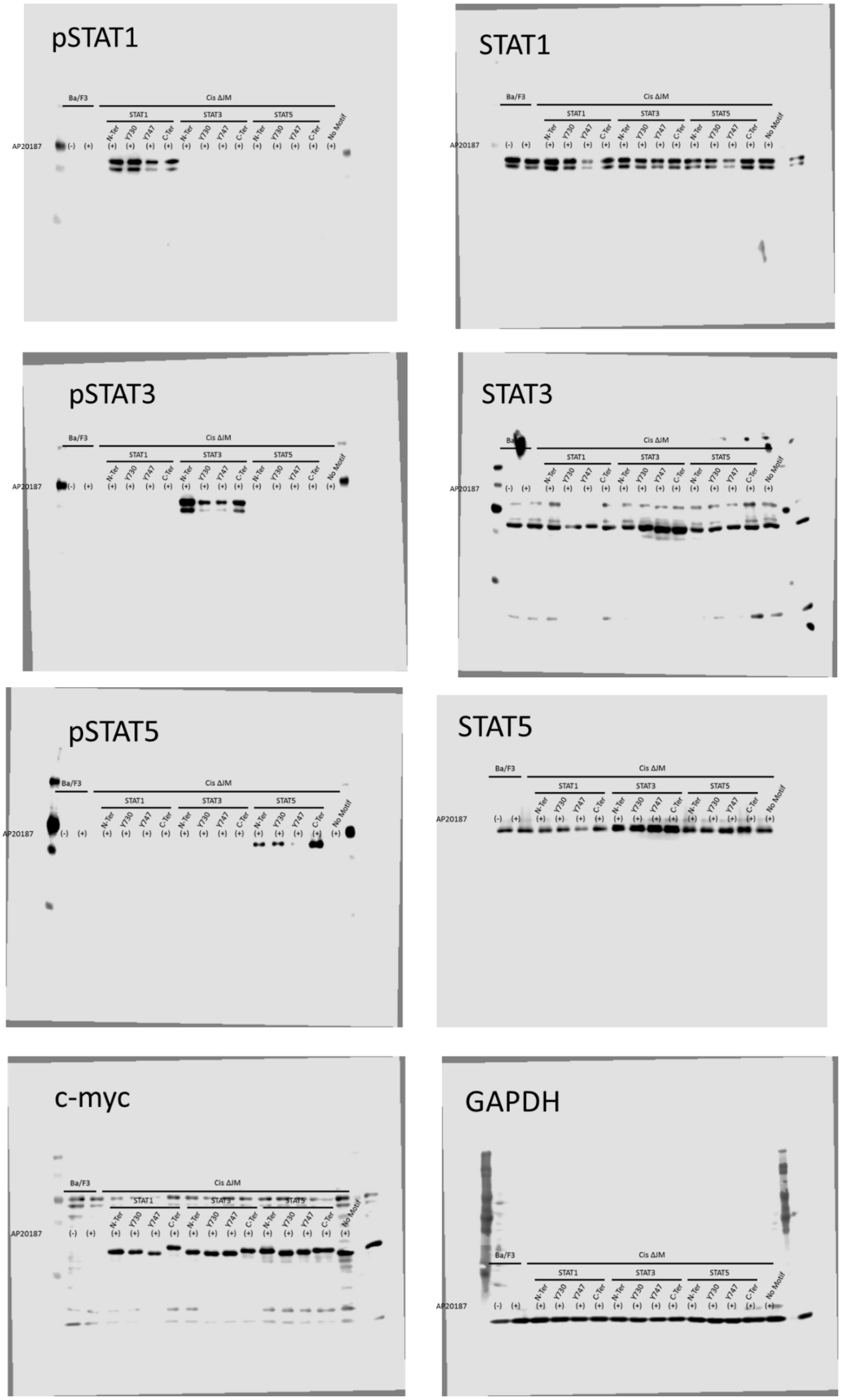

Supplementary Figure 16

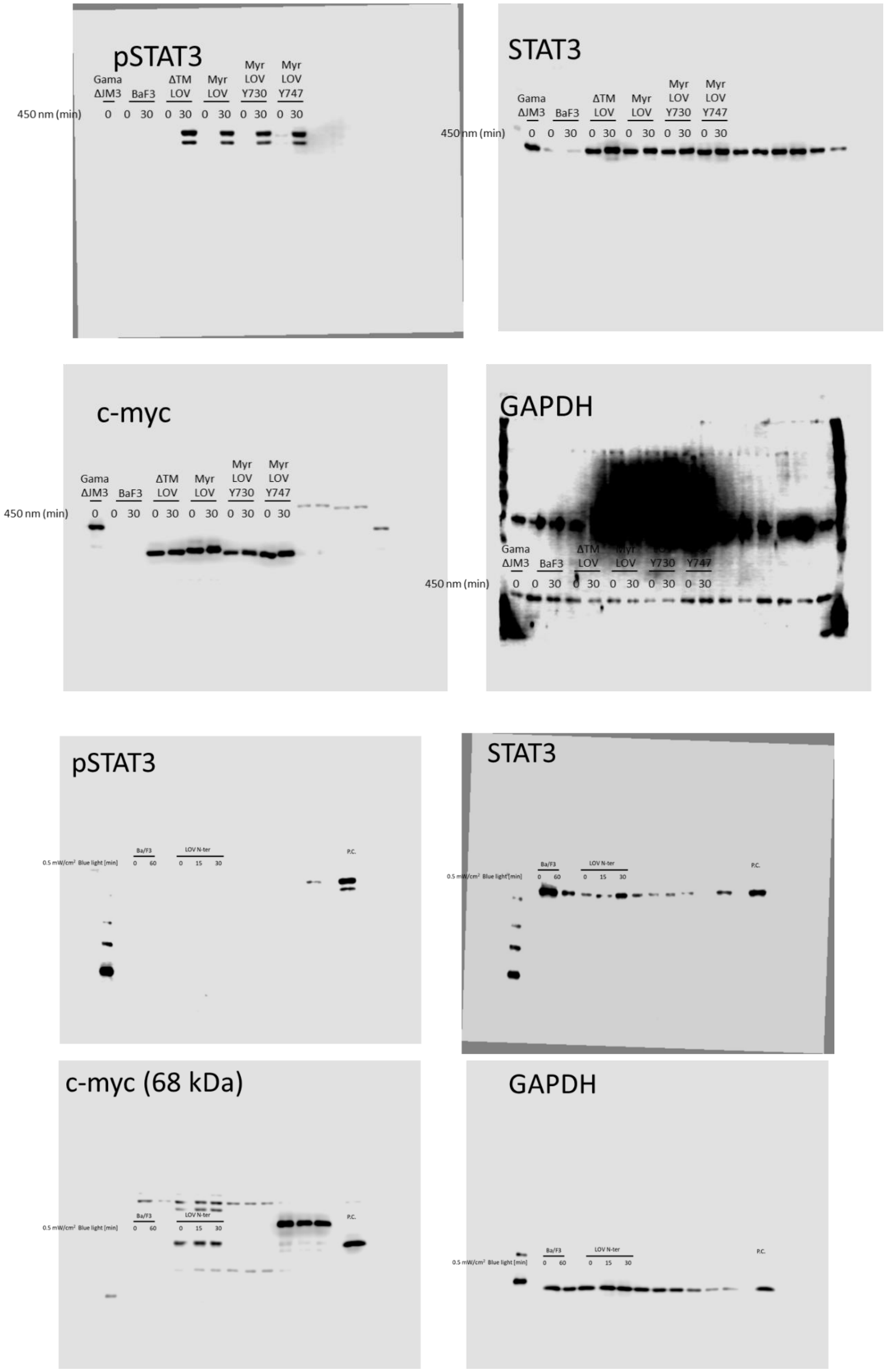

Supplement: Supplementary file 1 — Supplementary Information [file 42003_2021_2287_MOESM1_ESM.pdf]
