# Supplementary material for: Reprogramming signal transduction through a designer receptor tyrosine kinase
Source: Commun Biol. 2021 Jun 17;4:752. doi: 10.1038/s42003-021-02287-8 (PMC8211861; doi:10.1038/s42003-021-02287-8)
Supplement: Supplementary file 2 — Description of Supplementary Files [file 42003_2021_2287_MOESM2_ESM.pdf]

Description of Additional Supplementary Files

File name: Supplementary Data 1

Description: Source data for Fig. 4.
