# Supplementary material for: Reprogramming signal transduction through a designer receptor tyrosine kinase
Source: Commun Biol. 2021 Jun 17;4:752. doi: 10.1038/s42003-021-02287-8 (PMC8211861; doi:10.1038/s42003-021-02287-8)
Supplement: Supplementary file 4 — Reporting Summary [file 42003_2021_2287_MOESM4_ESM.pdf]

nature research

Corresponding author(s): Masahiro Kawahara

Last updated by author(s): May 7, 2021

Reporting Summary

Nature Research wishes to improve the reproducibility of the work that we publish. This form provides structure for consistency and transparency in reporting. For further information on Nature Research policies, see our Editorial Policies and the Editorial Policy Checklist.

Statistics

For all statistical analyses, confirm that the following items are present in the figure legend, table legend, main text, or Methods section.

n/a	Confirmed	
☐	☒	The exact sample size (*n*) for each experimental group/condition, given as a discrete number and unit of measurement
☒	☐	A statement on whether measurements were taken from distinct samples or whether the same sample was measured repeatedly
☒	☐	The statistical test(s) used AND whether they are one- or two-sided<br>*Only common tests should be described solely by name; describe more complex techniques in the Methods section.*
☒	☐	A description of all covariates tested
☒	☐	A description of any assumptions or corrections, such as tests of normality and adjustment for multiple comparisons
☒	☐	A full description of the statistical parameters including central tendency (e.g. means) or other basic estimates (e.g. regression coefficient) AND variation (e.g. standard deviation) or associated estimates of uncertainty (e.g. confidence intervals)
☒	☐	For null hypothesis testing, the test statistic (e.g. F, t, r) with confidence intervals, effect sizes, degrees of freedom and P value noted<br>*Give P values as exact values whenever suitable.*
☒	☐	For Bayesian analysis, information on the choice of priors and Markov chain Monte Carlo settings
☒	☐	For hierarchical and complex designs, identification of the appropriate level for tests and full reporting of outcomes
☒	☐	Estimates of effect sizes (e.g. Cohen's *d*, Pearson's *r*), indicating how they were calculated

Our web collection on statistics for biologists contains articles on many of the points above.

Software and code

Policy information about availability of computer code

Data collection	No software was used
Data analysis	No software was used

For manuscripts utilizing custom algorithms or software that are central to the research but not yet described in published literature, software must be made available to editors and reviewers. We strongly encourage code deposition in a community repository (e.g. GitHub). See the Nature Research guidelines for submitting code & software for further information.

Data

Policy information about availability of data

All manuscripts must include a data availability statement. This statement should provide the following information, where applicable:

- Accession codes, unique identifiers, or web links for publicly available datasets
- A list of figures that have associated raw data
- A description of any restrictions on data availability

We declare that the data supporting the findings of this study are available within the paper and its supplementary information files.

Field-specific reporting

Life sciences study design

All studies must disclose on these points even when the disclosure is negative.

Sample size	We empirically determined the sample size according to previous researches.
Data exclusions	No data were excluded from the analysis.
Replication	All attempts at application were successful.
Randomization	Randomized allocation was not relevant because all samples were treated similarly and no bias was expected.
Blinding	Blinding was not relevant because all samples were treated similarly and no bias was expected.

Reporting for specific materials, systems and methods

We require information from authors about some types of materials, experimental systems and methods used in many studies. Here, indicate whether each material, system or method listed is relevant to your study. If you are not sure if a list item applies to your research, read the appropriate section before selecting a response.

Materials & experimental systems

n/a	Involved in the study	
☐	☒	Antibodies
☐	☒	Eukaryotic cell lines
☒	☐	Palaeontology and archaeology
☒	☐	Animals and other organisms
☒	☐	Human research participants
☒	☐	Clinical data
☒	☐	Dual use research of concern

Methods

n/a	Involved in the study	
☒	☐	ChIP-seq
☒	☐	Flow cytometry
☒	☐	MRI-based neuroimaging

Antibodies

Antibodies used

Phospho-STAT1 (Tyr701) Antibody Cell Signaling Technology #9171S
STAT1 Antibody Cell Signaling Technology #9172
Phospho-STAT3 (Tyr705) (D3A7) Rabbit mAb Cell Signaling Technology #9145L
STAT3 (C-20) rabbit polyclonal IgG Santa Cruz Biotechnology sc-482
p-STAT5 (Y694) Rabbit Ab Cell Signaling Technology #9351S
STAT5 (c-17) rabbit polyclonal IgG Santa Cruz Biotechnology sc-835
Rabbit anti-c-myc Antibody affinity purified BETHYL A190-105A
GAPDH (FL-335) Santa Cruz Biotechnology sc-25778

Validation

1. Phospho-STAT1 (Tyr701) Antibody Cell Signaling Technology #9171S
https://www.cellsignal.jp/datasheet.jsp?productId=9171&images=1

2. STAT1 Antibody Cell Signaling Technology #9172
https://www.cellsignal.jp/datasheet.jsp?productId=9172&images=1

3. Phospho-STAT3 (Tyr705) (D3A7) Rabbit mAb Cell Signaling Technology #9145L
https://www.cellsignal.jp/datasheet.jsp?productId=9145&images=1

4. STAT3 (C-20) rabbit polyclonal IgG Santa Cruz Biotechnology sc-482
https://datasheets.scbt.com/sc-482.pdf

5. p-STAT5 (Y694) Rabbit Ab Cell Signaling Technology #9351S
https://www.cellsignal.jp/datasheet.jsp?productId=9351&images=1

6. STAT5 (c-17) rabbit polyclonal IgG Santa Cruz Biotechnology sc-835
https://datasheets.scbt.com/sc-835.pdf

7. Rabbit anti-c-myc Antibody affinity purified BETHYL A190-105A
https://www.bethyl.com/product/pdf/A190-105A.pdf

8. GAPDH (FL-335) Santa Cruz Biotechnology sc-25778

https://datasheets.scbt.com/sc-25778.pdf

Eukaryotic cell lines

Policy information about cell lines

Cell line source(s)	Ba/F3 cell line was purchased from RIKEN Cell Bank, Tsukuba, Japan; #RCB0805. Plat-E cell line was a generous gift from Dr. Toshio Kitamura (The University of Tokyo).
Authentication	Ba/F3 cell line was authenticated by the corresponding distributor. Plat-E cell line we used was not authenticated.
Mycoplasma contamination	The cell lines was not tested for microplasma contamination.
Commonly misidentified lines (See ICLAC register)	We did not use commonly misidentified lines.
